# Evidence against independence of peripheral latencies and central threshold in temporal-order perception

**DOI:** 10.3758/s13423-025-02797-0

**Published:** 2026-02-17

**Authors:** Paul Kelber, Rolf Ulrich

**Affiliations:** Department of Psychology, University of Tübingen, Schleichstraße 4, 72076 Tübingen, Germany

**Keywords:** Temporal-order judgment, Simultaneity judgment, Stimulus modality, Stimulus intensity

## Abstract

Independent-channels models of temporal-order and simultaneity perception assume that a central (amodal) timing mechanism compares the arrival times of incoming sensory signals independently of peripheral (modal) events. This implies that peripheral latencies and central threshold are additive. The present study tested whether the central threshold is invariant to intramodal versus intermodal stimulation (Experiment 1) and strong versus weak intermodal stimulation (Experiment 2). The central threshold was isolated via the distance between the two psychometric functions in the ternary-response task, which does not depend on peripheral latencies under the additivity assumption. In Experiment 1, the central threshold was estimated to be significantly higher for intermodal stimuli (light and sound) than for intramodal stimuli (two lights) for all subjects across the entire practice curve (20 one-hour sessions) and also at the group level. In Experiment 2, the estimates of the central threshold were significantly higher for weak intermodal stimuli (dim light and soft sound) than for strong ones (bright light and loud sound) for most subjects and at the group level. These violations of threshold invariance in both experiments provide converging evidence against the independence of peripheral latencies and central threshold, and thus challenge the dominant class of theoretical models of temporal-order and simultaneity perception.

**Supplementary Information:**

The online version contains supplementary material available at 10.3758/s13423-025-02797-0.

## Introduction

Our cognitive architecture imposes formidable limitations on our ability to discriminate events in time. This is not news, but one of the first insights of psychology: Wundt (1862, 1874) concluded from his famous complication experiment that a light must precede a sound by about 1/8 of a second for the two stimuli to arrive in consciousness simultaneously. Such constant errors reflect limits in the accuracy (i.e., unbiasedness) of temporal-order perception. Inaccurate temporal-order perception is readily explained by differences in peripheral latencies (i.e., “perceptual latencies” or “perception lags”; e.g., Gibbon & Rutschmann, 1969). However, in addition, a variable error limits the precision (i.e., consistency) of temporal-order perception. Experimental studies of this variable error, in particular, had a great impact on psychological theorizing about the mechanisms underlying temporal-order perception.

Historically, the seminal studies by Hirsh (1959) and Hirsh and Sherrick (1961) lent credence to and popularized the idea that the perceived order of different stimuli (e.g., light and sound) is determined by a single central instance in the brain. These studies examined the temporal-order threshold—operationalized as the smallest stimulus-onset difference *d* required for 75% accuracy in a temporal-order judgment (TOJ) task—for various stimulus pairs. In the TOJ task, one must decide which of two stimuli appeared first, while *d* is varied across trials. Hirsh (1959) found a temporal-order threshold of about 20 ms, independent of whether the to-be-ordered sounds differed in frequency, bandwidth, or duration. Hirsh and Sherrick (1961) extended these findings to other intramodal and intermodal stimulus pairs. Specifically, similar temporal-order thresholds were obtained for visual stimuli displayed at different spatial locations, auditory stimuli played in different ears, and tactile stimuli applied to different fingers. Most intriguingly, the temporal-order thresholds were also virtually identical ($\sim \,20$ ms) for different intermodal stimulus pairs (auditory–visual, auditory–tactile, and visual–tactile).

These strikingly consistent results “point to some kind of time-organizing system that is both independent of and central to the sensory mechanisms” (Hirsh & Sherrick, 1961, p. 431). As summarized below, most theoretical models proposed ever since adhere to this postulate by assuming that temporal-order perception involves a central (amodal) timing mechanism in the brain that compares the arrival times of incoming signals from peripheral channels (sensory modalities). Accordingly, the precision of temporal-order perception is limited not only by the variability in the peripheral latencies, but also by the temporal resolution of the central comparator.

In the 1950s to 1970s, several models linked Ira Hirsh’s postulate to perceptual-moment theory (von Baer, 1862; see also Kelber & Ulrich, 2025b), according to which subjective experience consists of a series of atomic temporal intervals (“moments”). Thus, two stimuli are perceived as ordered if their peripheral signals arrive at a central location in different moments, but as simultaneous if their arrivals fall within the same moment. This idea unites, among others, the autonomous-moments model (Stroud, 1956), the triggered-moments model (Pöppel, 1970; Venables, 1960), the moving-moment model (Allport, 1968), and attention-switching models (Allan, 1975a; Allan & Kristofferson, 1974; Kristofferson, 1967a, b).

Sternberg and Knoll (1973) have shown that many such models are special cases of a general *independent-channels model*. This model assumes that the sensory signals elicited by the stimuli travel through separate peripheral channels, and that their arrival-time difference at a central location is compared to a threshold (decision rule). To date, many more models have been proposed that supplement these basic assumptions with additional features such as variable decision criteria or moment durations (Kelber & Ulrich, 2025b; Sternberg et al., in press; Ulrich 1987; Yarrow et al., 2011; Yarrow et al., 2023), finger errors and attentional lapses (García-Pérez & Alcalá-Quintana, 2012a, b), separate centers for determining simultaneity/successiveness and temporal order (Jaśkowski, 1991b), or two thresholds for detecting successiveness and temporal order (Kelber & Ulrich, 2024; Sternberg et al., in press). Some of these models provide an excellent account of the shapes of the psychometric functions observed in various timing-judgment tasks (Kelber & Ulrich, 2024, 2025a; Sternberg et al., in press).

The architecture of these independent-channels models is based on the idea that a central timing mechanism compares the arrival-time difference to a threshold independently of peripheral events. Accordingly, the central threshold should be unaffected by aspects of peripheral stimulation, such as stimulus modality and intensity (*threshold-invariance assumption*). Although some models that incorporate the assumption of independent peripheral and central processing—along with other features—fit behavioral data well, it remains largely unclear whether this core assumption is empirically adequate on its own, as elaborated next.

The threshold-invariance assumption seems to be in line with the observation of a common temporal-order threshold across intramodal and intermodal stimulus pairs (Hirsh, 1959; Hirsh & Sherrick, 1961). However, several other studies revealed a less uniform picture, in which the temporal-order threshold in TOJ tasks varied among different stimulus pairs (e.g., Fink et al., 2005, 2006; Fostick & Babkoff 2013, 2017; Fostick et al., 2019; McFarland et al., 1998; Oatley et al., 1969; Swisher & Hirsh 1972; Tiippana & Salmela, 2018). Likewise, studies using simultaneity judgment (SJ; “simultaneous or successive?”) tasks also observed different successiveness thresholds across stimulus pairs (e.g., Foucher et al., 2007; Virsu et al., 2008; see also Exner, 1875).

One recurring account of these mixed results emphasizes that in contrast to several later studies, Hirsh (1959) and Hirsh and Sherrick (1961) tested highly trained subjects (Broadbent & Ladefoged, 1959; Gengel & Hirsh, 1970; Hirsh & Fraisse, 1964; Sherrick, 1969). This may hold relevance because thresholds have often been found to decrease with practice, both in TOJ tasks (e.g., Fostick & Babkoff, 2013; Gengel & Hirsh, 1970; Stevenson et al., 2013; Thor, 1968; Zhu et al., 2023) and SJ tasks (e.g., Horsfall et al., 2021a; Powers et al., 2009; Stevenson et al., 2013; Virsu et al., 2008; Zhu et al., 2023). Perhaps the thresholds only converge to a common precision level after suboptimal, stimulus-dependent decision strategies have been abandoned during extensive practice. Conversely, it is also conceivable that the thresholds start at a common precision level, but become increasingly sensitive to stimulus-dependent cues with continued practice. In any case, intramodal and intermodal thresholds should be monitored over the course of extensive practice to systematically test the threshold-invariance assumption.

A more fundamental problem with these previous measurements of temporal-order and successiveness thresholds is that they did not isolate the threshold in the central timing mechanism. Rather, they measured the steepness of the psychometric function in the TOJ task or the width of the psychometric function in the SJ task, both of which can be affected not only by the central threshold but also by the variability in the peripheral latencies. But since latency variability may be influenced by the type of stimulation, “compound measures” of the variable error do not allow for direct conclusions about the central timing mechanism (Sternberg & Knoll, 1973). Although the peripheral and central sources of the variable error can be separated in binary-response TOJ and SJ tasks, this requires specific distributional assumptions (e.g., Kelber & Ulrich, 2025b). Such commitments, however, prevent a general test of the threshold-invariance assumption described above.

By contrast, temporal judgments with more than two response alternatives allow a general test of threshold invariance. This is because they produce multiple psychometric functions, whose distance does not depend on peripheral latencies (Schwarz, 1988; Sternberg et al., in press; Ulrich, 1987). In the ternary-response task, one indicates for two stimuli *x* (e.g., light) and *y* (e.g., sound), which are physically separated by the stimulus-onset difference $d = t_y - t_x$, whether *x* appeared before *y* (*xy*), whether *y* appeared before *x* (*yx*), or whether *x* and *y* appeared simultaneously (*si*). This ternary response format, which combines TOJ and SJ, generates two psychometric functions, a right function $F_\text {R}(d) = P(xy\,|\,d)$ and a left function $F_\text {L}(d) = P(xy\,|\,d) + P(si\,|\,d) = 1 - P(yx\,|\,d)$. One advantage of this task is that the possibility to indicate simultaneity eliminates potential order-guessing biases in the case of perceived simultaneity. For example, this has proven useful in prior-entry research (Jaśkowski, 1993; Stelmach & Herdman, 1991; for other applications of the ternary-response task, see, e.g., Jaśkowski, 1991a; van Eijk et al., 2008).

How do people make ternary judgments about temporal order and simultaneity? Assume that the arrival-time difference at the central timing mechanism, $\mathbf {\Delta A} = \textbf{A}_y - \textbf{A}_x$, which is the sum of the latency difference $\boldsymbol{\Delta }\textbf{L} = \textbf{L}_y - \textbf{L}_x$ and the stimulus-onset difference $d = t_y - t_x$, is compared to the central threshold $\textbf{C}$. Thus, *xy* results if $\boldsymbol{\Delta }\textbf{A} \ge \textbf{C}$, *yx* if $\boldsymbol{\Delta }\textbf{A} \le -\textbf{C}$, and *si* if $|\boldsymbol{\Delta }\textbf{A}| < \textbf{C}$. As shown by Ulrich (1987), $F_{\text {R}}$ is obtained by adding the central threshold $\textbf{C}$ to the difference between the peripheral latencies of the two stimuli, $\boldsymbol{\Delta }\textbf{L}$, whereas $\textbf{C}$ has to be subtracted from $\boldsymbol{\Delta }\textbf{L}$ to arrive at $F_{\text {L}}$. Thus, the right function is centered at the location $\text {E}(F_\text {R}) = \text {E}(\boldsymbol{\Delta }\textbf{L} + \textbf{C})$, and the left function at $\text {E}(F_\text {L}) = \text {E}(\boldsymbol{\Delta }\textbf{L} - \textbf{C})$.

Importantly, this prediction builds only on the additivity of the peripheral latencies and the central threshold, which reflects precisely the threshold-invariance assumption to be tested. Without further assumptions, it follows that the distance between the two psychometric functions in the ternary-response task depends solely on the central threshold, as the contribution of the peripheral latencies cancels out. Formally,

1 $$\begin{aligned} \text {E}(F_\text {R}) - \text {E}(F_\text {L})&= \text {E}(\boldsymbol{\Delta }\textbf{L} + \textbf{C}) - \text {E}(\boldsymbol{\Delta }\textbf{L} - \textbf{C}) \nonumber \\&= \text {E}(\boldsymbol{\Delta }\textbf{L}) + \text {E}(\textbf{C}) - \text {E}(\boldsymbol{\Delta }\textbf{L}) + \text {E}(\textbf{C}) \nonumber \\&= 2 \cdot \text {E}(\textbf{C}). \end{aligned}$$

Independent-channels models thus predict that the central threshold, measured as the half distance between the psychometric functions in the ternary-response task, is invariant to changes in peripheral stimulation, assuming that the central threshold is stimulus-independent. This prediction was systematically tested in the present study.

To our knowledge, the prediction above has not yet been tested through modality variations, such as by presenting intramodal versus intermodal stimulus pairs. By contrast, there is evidence against the invariance of the central threshold to visual stimulus intensity (Ulrich, 1987). When manipulating whether two vertically displaced lights were either both dim or both bright, the distance between the psychometric functions in the ternary-response task decreased reliably with increasing brightness for each of three subjects. However, this violation of threshold invariance may be explained by assuming that the timing of spatially separated lights is determined by the presence and direction of apparent motion (e.g., Ono, 2025; Yamamoto & Kitazawa, 2001). Then the “simultaneity span” should shrink with increasing visual stimulus intensity, given that brighter light flashes strengthen apparent motion (Allport, 1970).

Such confounds can be minimized by testing threshold invariance with intermodal stimuli: Is the central threshold the same when judging the timing of (1) a dim light and a soft sound or (2) a bright light and a loud sound? We are unaware of any pertinent data that isolate the central threshold via the latency-free distance between the psychometric functions in the ternary-response task. However, as described above, this method is crucial for decomposing the variable error into its peripheral and central sources, considering that peripheral latencies decrease in mean—and thus most probably also in variability—with increasing stimulus intensity (e.g., Boenke et al., 2009; Horsfall et al., 2021b; Horsfall et al., 2024; Leone & McCourt, 2015; Neumann & Niepel, 2024; Neumann et al., 1992; Roufs, 1963; Smith, 1933). This makes it difficult to draw conclusions about the central timing mechanism from observations that weaker stimuli lead to higher threshold estimates in binary-response TOJ or SJ tasks (e.g., Krueger Fister et al., 2016; Terao et al., 2008).

In the present study, we tested the additivity of peripheral latencies and central threshold assumed by independent-channels models of temporal-order and simultaneity perception. The threshold-invariance assumption was evaluated by comparing the estimates of the central threshold obtained for intramodal versus intermodal stimuli (Experiment 1) and strong versus weak intermodal stimuli (Experiment 2) in the ternary-response task. Independent-channels models predict that the central threshold is unaffected by these manipulations of stimulus modality and intensity. Precise data for each subject allowed us to test for threshold invariance at the individual level and with several independent replications per experiment (Smith & Little, 2018).

## Experiment 1

This experiment tested whether the central threshold is invariant to intramodal versus intermodal stimulation. Five subjects judged the timing of visual and audio-visual stimuli in a ternary response format. Alternating mini-blocks featured (1) two light flashes or (2) one flash and one click. Each subject completed 20 one-hour sessions (20,000 trials in total) to address the concern that central thresholds converge or diverge during extensive practice.

### Method

#### Subjects

The four naïve subjects AN (male, right-handed, 23 years), MS (male, right-handed, 24 years), KS (male, right-handed, 23 years), and WF (male, right-handed, 22 years), as well as the first author (subject PK, male, left-handed, 27 years) participated in 20 one-hour sessions each. For Experiments 1 and 2, all subjects reported normal or corrected-to-normal vision and audition, and everyone except the first author received course credit or German minimum wage (12.41€ per hour in 2024).

The preregistered sample size of five subjects was determined to strike a balance between precise threshold estimation across the practice curve at the fine-grained individual level, the possibility of additional group-level analyses, and the practical feasibility of data collection. This sample size was similar to those in many previous TOJ/SJ experiments with individual-level analyses (e.g., Allan, 1975a, b; Jaśkowski, 1993; Kelber and Ulrich, 2025a; Tünnermann & Scharlau, 2018, 2021; Ulrich 1987; Yarrow et al., 2016), and also ensured a statistical power of above 80% to detect the observed effect sizes of $d_z =$ 1.79-−1.89 at the group level (as calculated with G*Power, Version 3.1.9.7; Faul et al., 2007). In Experiment 2, a sample size of 10 subjects was preregistered to enhance the generalizability of the findings. The observed large effect sizes and the highly consistent results across subjects, practice levels, threshold-estimation methods, and experiments together suggest that the conclusions drawn about the basic timing mechanisms under investigation will generalize beyond the tested samples.

#### Apparatus and stimuli

The experiment was conducted inside a sound- and light-attenuated booth. Stimulus presentation and response recording were controlled by a Fujitsu Esprimo P910 E85+ computer (Windows 10, 64 bit) located outside the booth, which was equipped with a Nvidia GeForce GT 710 graphics card and a VizGiz USB sound card. This computer ran a custom Python script with code based on the PsychoPy library (Peirce et al., 2019). Visual stimuli were circular patches of light (diameter: $1^{\circ }$ of visual angle) displayed against a dark background via a ViewSonic XG2431 IPS-LCD monitor (120 Hz, 24 inch, $1920 \times 1080$ pixels). For intermodal stimuli, the light was presented in the screen center; for intramodal stimuli, two lights were presented $4^{\circ }$ of visual angle above and below the screen center. The lights had a luminance of 4.8 cd/m$^2$, as measured with a Gigahertz Optik P-9201-TF photometer from the subject’s position. The subject viewed these stimuli from a chinrest placed about 53 cm in front of the screen. Auditory stimuli were white noise presented via two Creative GigaWorks T20 Series II loudspeakers. These loudspeakers were located left and right of the monitor and about 60 cm away from the subject’s ears. The presented sounds had a sound pressure level of 62.6 dB(A), as measured with a Casella CEL 275 sound level meter from the subject’s position. Both light and sound were presented for approximately 8 ms, considering that short stimuli have been found to yield more precise order discrimination compared to long or response-terminated stimuli (e.g., Oatley et al., 1969) and thus may be more informative about optimal timing performance.

The duration and relative timing of the stimuli were validated using an external Black Box ToolKit v2 (Plant et al., 2004). Specifically, each stimulus duration and each stimulus-onset difference *d* was measured 20 times. According to these measurements, the light lasted 8.4 ms ($SD = 0.1$ ms) and the sound 8.3 ms ($SD = 0.8$ ms). Furthermore, each of the 25 *d* values was delivered with both sub-millisecond accuracy (mean bias: 0.0 ms, range: $-0.3$−0.3 ms) and millisecond precision (mean variability: 0.9 ms, range: 0.6−1.3 ms) for audio-visual stimuli, and with millisecond accuracy (mean bias: 1.7 ms, range: 1.7−1.8 ms) and sub-millisecond precision (variability always 0.1 ms) for visual stimuli.

Responses were collected via a button box with three horizontally arranged response buttons. For audio-visual (visual) stimuli, subjects were instructed to press the left button if the visual (top) stimulus appeared first, the middle button if the stimuli appeared simultaneously, and the right button if the auditory (bottom) stimulus appeared first. Subjects were instructed to correct their response if they made a finger error, and to press one button twice if they had an attentional lapse (cf. Kelber & Ulrich, 2025a).

#### Procedure

Each experimental trial started with a 500 ms empty interval of darkness and silence, followed by the first stimulus. The second stimulus was presented 0, 16.$\bar{6}$, ..., or 200 ms after the onset of the first stimulus. The offset of the second stimulus then initiated another empty interval that lasted until the end of the trial. Finger errors and attentional lapses had to be reported in a 1,000 ms interval after the first, unspeeded response. If the subject again pressed a button in this interval (to correct a finger error or to indicate an attentional lapse), another 1,000 ms interval began. A trial ended as soon as one post-response interval had elapsed without any further response.

The stimulus-onset difference *d* ranged from $-200$ ms to 200 ms in steps of 16.$\bar{6}$ ms (cf. Kelber & Ulrich, 2025a). Each of the 25 *d* values was presented twice per block, with the resulting 50 trials arranged in random order. One session comprised 20 blocks (i.e., $50 \times 20 = 1{,}000$ trials), in which the stimuli alternated between visual and audio-visual (e.g., visual block $\rightarrow$ audio-visual block $\rightarrow$ visual block $\rightarrow$ ...). These alternating mini-blocks of visual and audio-visual stimuli were employed to reduce potential criterion shifts between the two conditions, after pilot testing indicated that unpredictable trial-by-trial modality changes were disruptive to task performance and thus impractical for Experiment 1. The subject could take a self-paced break between two blocks. No feedback was provided throughout the experiment.

**Fig. 1:**
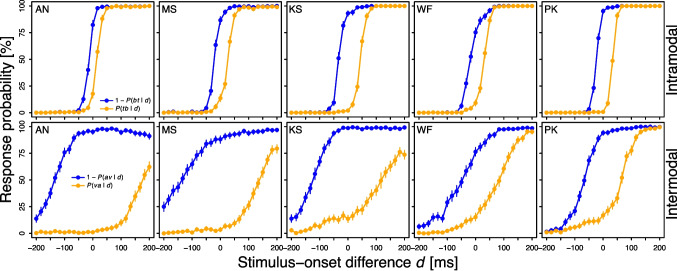
Psychometric functions observed in Experiment 1. For intramodal stimuli, *tb* (*bt*) refers to the judgment that the top visual stimulus *t* appeared before (after) the bottom visual stimulus *b*. For intermodal stimuli, *va* (*av*) refers to the judgment that the visual stimulus *v* appeared before (after) the auditory stimulus *a*. Error bars reflect bootstrapped 95% CIs

**Table 1:** Threshold estimates in Experiment 1

		Subject				
Estimation	Threshold	AN	MS	KS	WF	PK
Non-parametric	Intermodal	143 [141, 145]	123 [121, 125]	111 [109, 113]	62 [60, 63]	59 [57, 60]
	Intramodal	14 [13, 15]	23 [22, 24]	37 [36, 38]	21 [20, 22]	28 [27, 29]
	*Difference*	129 [127, 131]	100 [98, 103]	74 [73, 77]	41 [39, 42]	31 [29, 32]
Parametric	Intermodal ($c_{su}$)	149 [147, 151]	131 [128, 133]	115 [112, 117]	62 [61, 64]	59 [57, 60]
	Intramodal ($c_{su}$)	14 [13, 15]	22 [21, 23]	36 [35, 37]	21 [20, 22]	28 [27, 29]
	*Difference* ($c_{su}$)	135 [133, 137]	109 [106, 111]	79 [76, 81]	41 [40, 43]	31 [29, 32]
	Intermodal ($c_o$)	164 [161, 166]	146 [143, 149]	134 [131, 136]	68 [66, 71]	72 [70, 74]
	Intramodal ($c_o$)	16 [14, 17]	24 [23, 25]	38 [37, 39]	27 [26, 28]	29 [28, 30]
	*Difference* ($c_o$)	148 [145, 151]	122 [118, 124]	96 [93, 99]	41 [38, 44]	43 [41, 45]

The values in square brackets represent the lower and upper limit of the bootstrapped 95% CI. All values were rounded to the nearest millisecond. $c_{su}$: successiveness threshold, $c_o$: order threshold

Each subject completed 20 sessions on separate days at similar times. This resulted in a total of $1{,}000 \times 20 = 20{,}000$ trials per subject, of which the first 100 trials (the first two blocks of the first session) were considered practice. Hence, each *d* value was presented in 400 trials (minus two practice trials) for each of the two stimulus conditions.

#### Analysis

We excluded practice trials, premature responses (RTs $< 150$ ms; mean across subjects: 0.2% of the trials, range: 0.0−0.8%), delayed responses (RTs $> 5{,}000$ ms; mean: 0.1%, range: 0.0−0.2%), and attentional lapses (double-clicks; mean: 0.2%, range: 0.1−0.5%), and we accounted for finger errors (response corrections; mean: 1.0%, range: 0.0−3.2%).

To obtain a distribution-free measure of the distance between the two psychometric functions in the ternary-response task, the modified Spearman-Kärber method was used (for details, see Miller & Ulrich, 2001; Sternberg et al., 1982). Following Ulrich (1987), we estimated the mean of each function and defined the central threshold as the half difference between the two mean values. Although distribution-free, the Spearman-Kärber method requires specification of the *d* values at which the function reaches 0 and 1 (Experiment 1: $\pm 225$ ms, Experiment 2: $\pm 400$ ms). However, explorations with other values suggested the results of the threshold-invariance tests are generally robust to different placements of these start and end points.

For the Spearman-Kärber method, the observed psychometric functions also had to be monotonized to obtain maximum-likelihood estimates of their mean (Ayer et al., 1955). However, several TOJ/SJ models allow for non-monotonic psychometric functions in the ternary-response task (García-Pérez & Alcalá-Quintana, 2012b; Jaśkowski, 1991b; Kelber & Ulrich, 2024; Sternberg et al., in press). Of these, the two-threshold model provided the best fit to observed data, and its specific assumptions are empirically the least inadequate (Kelber & Ulrich 2024, 2025a; see also Sternberg et al., in press). Thus, to cover the case that monotonicity is violated, we also parametrically tested for threshold invariance by fitting the two-threshold model. According to this model, the (approximately) Gaussian difference between the peripheral latencies of the two stimuli may reach the successiveness threshold $c_{su}$ but not the order threshold $c_o$, since $c_o \ge c_{su}$. In this case, temporal order must be guessed (for computational details, see Kelber & Ulrich, 2024). The application of this model allowed us to test the invariance assumption separately for the successiveness threshold and the order threshold, which often do not coincide empirically (e.g., Exner, 1875; García-Pérez & Alcalá-Quintana, 2015; Hirsh, 1959; Kelber & Ulrich, 2025a).

Both in the non-parametric (Spearman-Kärber method) and parametric analysis (two-threshold model), a percentile-bootstrap approach was used to draw statistical inferences at the individual level. Specifically, the thresholds and their difference were estimated for 1,000 bootstrap samples, and the 2.5% and 97.5% percentile of the resulting distribution of each quantity were considered the lower and upper limits of the 95% confidence interval (CI). Threshold invariance was said to be violated only if the bootstrapped 95% CI of the difference between the thresholds for intermodal and intramodal stimuli did not cover zero.

The Supplementary Material tabulates the individual data (Tables S1–14) and the individual proportions of corrected/excluded responses (Tables S15–16), and illustrates the fits of the two-threshold model (Figs. S1–7) for both experiments.

### Results and discussion

Fig. 1 shows the psychometric functions for intramodal and intermodal stimulation pooled across all 20 sessions for each subject in Experiment 1. For every subject, the two psychometric functions were noticeably further apart for intermodal stimuli than for intramodal stimuli. This was statistically substantiated (see Table 1): Threshold invariance was violated for each of the five subjects and three threshold estimation methods. Also at the group level, intermodal and intramodal stimuli led to significantly different estimates of the non-parametric threshold (intermodal: 100 ms, intramodal: 25 ms), $t(4) = 4.12$, $p =.015$, $d_z = 1.84$, parametric successiveness threshold (103 versus 24 ms), $t(4) = 4.00$, $p =.016$, $d_z = 1.79$, and parametric order threshold (117 versus 27 ms), $t(4) = 4.23$, $p =.013$, $d_z = 1.89$.

**Fig. 2:**
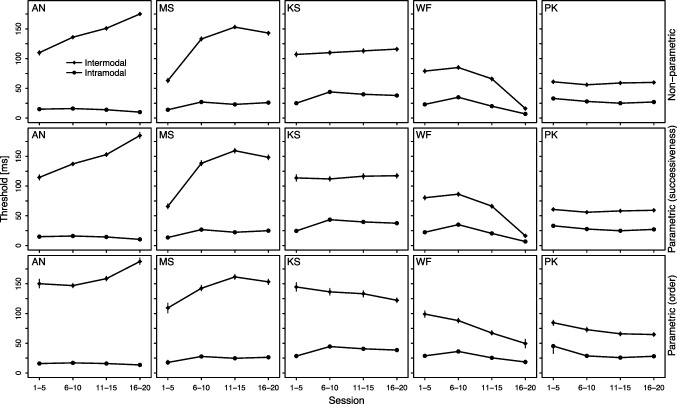
Practice curve of the intramodal and intermodal threshold in Experiment 1. Error bars reflect bootstrapped 95% CIs

Figure 2 presents the central thresholds in four practice levels, each comprising five of the 20 sessions. Intramodal thresholds were fairly stable, whereas intermodal thresholds exhibited interindividually different practice curves: increasing for AN and MS, approximately constant for KS and PK, and decreasing for WF. In agreement with the results from Yarrow et al. (2023), this suggests that the intermodal threshold can be sensitive to criterion shifts. However, most importantly, the intermodal threshold was significantly above the intramodal threshold across the entire practice curve for all subjects and threshold measures. Experiment 1 therefore provides consistent evidence against the threshold-invariance assumption of independent-channels models.

## Experiment 2

Experiment 2 controlled for two alternative explanations of the threshold-invariance violations in Experiment 1. First, the temporal order of two lights may have been determined using peripheral cues, such as the direction of apparent motion (Ono, 2025; Yamamoto & Kitazawa, 2001), which are typically less salient or even unavailable for intermodal stimuli. One could thus argue that independent-channels models are only applicable to intermodal comparisons and not to intramodal ones. However, it remains unclear whether the threshold-invariance assumption of these models holds for purely intermodal comparisons. This is because Experiment 1 in the present study compared thresholds for intramodal and intermodal stimuli, and Ulrich (1987) compared thresholds for strong and weak intramodal stimuli. Therefore, we cannot yet evaluate the prediction of independent-channels models that the central threshold does not depend on whether to-be-ordered intermodal stimuli are strong or weak. Experiment 2 thus tested whether the central threshold varies with intermodal stimulus intensity by presenting either a bright light with a loud sound or a dim light with a soft sound. Note that the *d* range was expanded from $\pm 200$ ms to $\pm 350$ ms to better capture the psychometric functions for these intermodal stimuli.

Second, intramodal versus intermodal stimulation varied block-wise in Experiment 1 because pilot testing indicated that trial-by-trial modality changes were too distracting to allow precise temporal judgments. The consistent threshold-invariance violations may thus have been due to criterion shifts, although this seems unlikely due to the alternating mini-blocks. However, to eliminate this potential confound, stimulus intensity varied unpredictably from trial to trial in Experiment 2.

Since the outcome of Experiment 1 was not systematically influenced by practice, we also reduced the number of sessions per subjects from 20 to five, allowing us to double the number of subjects from five to 10.

### Method

#### Subjects

Ten naïve subjects (six female, nine right-handed, mean age: 20 years, age range: 19–27 years) completed five one-hour sessions each. Two of them (FL and LF) also participated in one earlier session, which was deemed practice due to particularly imprecise judgments.

**Fig. 3:**
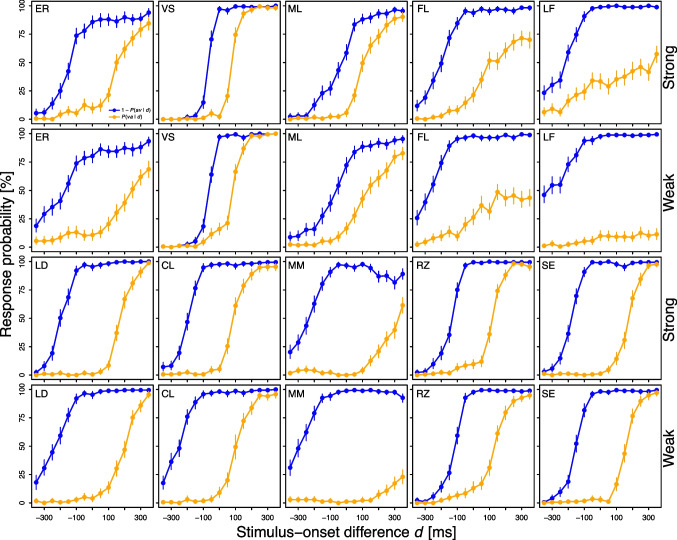
Psychometric functions observed in Experiment 2. The term *va* (*av*) refers to the judgment that the visual stimulus *v* appeared before (after) the auditory stimulus *a*. Error bars reflect bootstrapped 95% CIs

#### Apparatus, stimuli, and procedure

Each trial involved a light displayed in the screen center and a sound played via loudspeakers. The strong stimuli had a luminance of 10.6 cd/m$^2$ and a sound pressure level of 77.6 dB(A), as compared to 1.4 cd/m$^2$ and 43.2 dB(A) for the weak stimuli. These intensity levels were determined by a cross-modal matching procedure conducted by the first author. If these brightness and loudness increments were unequal, the locations of both psychometric functions should shift between strong and weak stimuli. However, it should not affect the distances between the psychometric functions, for which the contribution of the peripheral latencies cancels out (see Eq. 1). The tests of threshold invariance are thus robust to mismatching intensity levels.

The stimulus-onset difference *d* ranged from $-350$ ms to 350 ms in steps of 50 ms, following van Eijk et al. (2008). All *d* values were delivered with sub-millisecond accuracy (mean bias: 0.2 ms, range: 0.1−0.4 ms) and sub-millisecond precision (mean variability: 0.6 ms, range: 0.5−0.8 ms). Each block consisted of 60 trials (15 *d* values $\times$ 2 intensity levels $\times$ 2 repetitions), presented in random order. One session comprised 18 blocks (60 $\times$ 18 = 1,080 trials). The first block in the first session was considered practice. Across the five sessions per subject, each *d* value was presented in 180 trials (minus two practice trials) for each intensity condition.

#### Analysis

We excluded practice trials, a total of seven blocks due to technical issues, premature responses (RTs $< 150$ ms; mean: 0.1%, range: 0−0.2%), delayed responses (RTs $> 5{,}000$ ms; mean: 0.1%, range: 0−0.6%), lapses (double-clicks; mean: 1.1%, range: 0.0−4.8%), and we accounted for finger errors (response corrections; mean: 0.8%, range: 0.0−2.6%).

### Results and discussion

Fig. 3 shows the psychometric functions for strong and weak intermodal stimuli pooled across the five sessions per subject in Experiment 2. While less salient than in Experiment 1, it can be seen that for most subjects, the functions were further apart for weak compared to strong stimuli. This difference is most striking for MM and LF: Their psychometric functions were decently captured for strong stimuli, but barely for weak ones. This is bad news for the prospect of accurate threshold estimation in these latter cases, but also nicely illustrates that the distance between the functions depends on intermodal stimulus intensity.

**Table 2:** Threshold estimates in Experiment 2

		Subject				
Estimation	Threshold	ER	LD	VS	CL	ML
Non-parametric	Weak	167 [160, 172]	209 [203, 213]	69 [65, 73]	180 [175, 185]	104 [97, 110]
	Strong	133 [128, 139]	184 [179, 188]	72 [69, 75]	145 [140, 150]	82 [77, 87]
	*Difference*	34 [25, 41]	25 [18, 32]	$-3$ [$-8$, 3]	35 [28, 41]	22 [15, 29]
Parametric	Weak ($c_{su}$)	178 [170, 186]	212 [206, 218]	68 [64, 72]	182 [177, 189]	108 [102, 115]
	Strong ($c_{su}$)	135 [128, 140]	185 [180, 190]	71 [68, 75]	145 [140, 150]	84 [78, 89]
	*Difference* ($c_{su}$)	43 [33, 54]	27 [19, 35]	$-3$ [$-9$, 2]	37 [29, 46]	24 [16, 33]
	Weak ($c_o$)	265 [251, 281]	219 [213, 226]	84 [79, 89]	191 [186, 199]	141 [129, 152]
	Strong ($c_o$)	188 [179, 197]	189 [184, 194]	77 [74, 81]	154 [148, 161]	108 [99, 117]
	*Difference* ($c_o$)	77 [61, 95]	30 [22, 38]	7 [1, 13]	37 [29, 46]	33 [17, 47]
		MM	FL	RZ	LF	SE
Non-parametric	Weak	302 [298, 306]	218 [211, 226]	123 [118, 127]	296 [292, 303]	155 [151, 161]
	Strong	241 [236, 246]	168 [162, 174]	126 [123, 131]	191 [185, 198]	177 [172, 182]
	*Difference*	61 [54, 69]	50 [43, 58]	$-3$ [$-11$, 2]	105 [97, 116]	$-22$ [$-28$, $-15$]
Parametric	Weak ($c_{su}$)	345 [333, 359]	248 [239, 257]	122 [116, 127]	390 [378, 400]	156 [151, 160]
	Strong ($c_{su}$)	250 [243, 257]	180 [173, 188]	125 [120, 130]	211 [200, 220]	177 [172, 182]
	*Difference* ($c_{su}$)	95 [82, 111]	68 [55, 79]	$-3$ [$-10$, 4]	179 [164, 195]	$-21$ [$-28$, $-15$]
	Weak ($c_o$)	376 [360, 392]	301 [289, 316]	138 [132, 144]	422 [407, 437]	163 [158, 169]
	Strong ($c_o$)	304 [294, 316]	195 [185, 205]	135 [130, 140]	311 [295, 328]	184 [178, 189]
	*Difference* ($c_o$)	72 [52, 90]	106 [90, 123]	3 [$-6$, 11]	111 [89, 133]	$-21$ [$-27$, $-13$]

The values in square brackets represent the lower and upper limit of the bootstrapped 95% CI. All values were rounded to the nearest millisecond. $c_{su}$: successiveness threshold, $c_o$: order threshold

Table 2 allows for statistical inferences at the individual level. Threshold invariance was violated for eight of 10 subjects when considering non-parametric threshold estimates and parametric estimates of the successiveness threshold, and for nine of 10 subjects when considering parametric estimates of the order threshold. Apart from subject SE, these violations were always in the direction of a higher threshold for weak stimuli. In line with this, the threshold was significantly higher for weak versus strong stimuli at the group level. This held true for the non-parametric threshold (weak: 182 ms, strong: 152 ms), $t(9) = 2.63$, $p =.027$, $d_z = 0.83$, parametric successiveness threshold (201 versus 156 ms), $t(9) = 2.41$, $p =.039$, $d_z = 0.76$, and parametric order threshold (230 versus 184 ms), $t(9) = 3.23$, $p =.010$, $d_z = 1.02$. Experiment 2 thus provides further evidence against threshold invariance.

## General discussion

Independent-channels models of temporal-order and simultaneity perception assume that a central timing mechanism is only informed by the arrival times of the stimuli. The present study tested the assumption of these models that the central threshold is invariant to changes in peripheral stimulation. To this end, the central threshold was isolated via the distance between the two psychometric functions in the ternary-response task. This distance depends only on the central threshold and not on the peripheral latencies under the to-be-tested assumption of their additivity (see Eq. 1).

Precise individual data were collected in two multi-session experiments to evaluate whether the central threshold is sensitive to intramodal versus intermodal stimulation (Experiment 1) and to strong versus weak intermodal stimulation (Experiment 2). In Experiment 1, the central threshold was estimated to be significantly higher for intermodal versus intramodal stimulation across the entire practice curve (20 one-hour sessions) of each subject and also at the group level. Similarly, in Experiment 2, the central threshold was estimated to be significantly higher for weak versus strong intermodal stimulation across most subjects and at the group level. Taken together, Experiments 1 and 2 provide converging evidence against the threshold-invariance assumption. This challenges not only traditional perceptual-moment models (e.g., Allport , 1968; Pöppel, 1970; Sternberg & Knoll, 1973; Stroud, 1956), but also many current candidate models of temporal-order perception (e.g., García-Pérez & Alcalá-Quintana, 2012a; Kelber & Ulrich, 2024; Schneider & Bavelier, 2003; Sternberg et al., in press; Yarrow et al., 2011, 2023).

How generalizable are the present findings? One concern is that the results obtained with the ternary-response task may not generalize to other tasks, such as binary-response TOJ and SJ. The observed pattern might be task-specific, especially since is unclear whether TOJ, SJ, and ternary-response tasks engage the same underlying mechanisms (see also Spence & Parise, 2010, p. 365). Some experimental manipulations have different effects on the thresholds measured in TOJ and SJ tasks, such as the spatial distance between tactile stimuli (Geffen et al., 2000, Shore et al., 2005), and TOJ and SJ performance have partially distinct neural correlates (e.g., Binder, 2015; Miyazaki et al. 2016; Love et al., 2018). However, the present findings align with several previous model-based findings in other tasks. Cary et al. (2024) fitted two specific independent-channels models to SJs and found that peripheral-latency variability was predictive of the central threshold. Similarly, Kelber and Ulrich (2024, 2025a, 2025b) generally obtained higher model-based threshold estimates for intermodal than for intramodal stimuli across TOJ, SJ, and ternary-response tasks. While this suggests that the present findings extend to other tasks, these previous findings are limited to individual models with specific assumptions and therefore do not permit general conclusions about the additivity of peripheral latencies and central threshold. By contrast, the present evidence against threshold invariance is general in that it directly challenges additivity.

However, another concern is that the present findings might not generalize to other stimulus pairs and presentation methods. The only other evidence, which isolates the central threshold via the distance between the two psychometric functions in the ternary-response task, is the finding from Ulrich (1987) that the central threshold was estimated to be higher for weak than for strong visual stimuli. The present study extends this evidence to the often-debated comparison of intramodal (visual) versus intermodal (audio-visual) stimulation (e.g., Hirsh & Sherrick, 1961; Tiippana & Salmela, 2018), and to the comparison of strong versus weak intermodal (audio-visual) stimulation, which minimizes potential confounds by peripheral cues for judging temporal order. Nonetheless, conceptual replications with other stimulus pairs are warranted to determine the scope of the threshold-invariance violations. Future studies may also allow repeated presentations of the same stimulus pair in each trial. This procedure from Hirsh and Sherrick (1961) has been considered a possible explanation for their finding of a stimulus-independent temporal-order threshold (e.g., Gengel & Hirsh, 1970). The other possible explanation often discussed—extensive practice—now seems unlikely, as threshold invariance was consistently violated over the course of 20 sessions in Experiment 1.

How can independent-channels models account for the present findings? Importantly, the present findings do not rule out the possibility that peripheral latencies and central threshold are independent if additivity is only assumed to hold within any single trial. This would allow stimulus-dependent central thresholds, but also raise the question of how these could arise. Below we discuss a “variability-adaptation account” inspired by Cary et al. (2024), followed by a “multiple-oscillations account” borrowed from VanRullen and Koch (2003).

First, the central timing mechanism may somehow adapt to the variability in the peripheral latencies (Cary et al., 2024). This adaptation would have to result in higher thresholds for more variable latencies, such as for weaker or later integrated (intermodal) stimuli. However, it is unclear how the central timing mechanism could infer the variability in the peripheral latencies, given that its only inputs are the central arrival times of the stimuli. Perhaps the threshold (or the arrival-time difference) is updated from trial to trial according to the deviation between the arrival-time differences in the current and previous trial. Such a dynamic process would be reminiscent of internal-reference updating in duration discrimination (e.g., Dyjas et al., 2012; Ellinghaus et al., 2024; see also de Jong et al., 2021; Schumacher & Voss, 2023), and might also account for temporal-recalibration phenomena (e.g., Vroomen et al., 2004; see also Yarrow et al., 2015). But critically, even for block-wise stimulus manipulations (such as in Experiment 1), simulations with several updating formulas did not reveal any notable threshold-invariance violations. The variability-adaptation account therefore seems to lack a plausible mechanism.

Second, multiple stimulus-dependent oscillations may serve as the neural implementation of different central thresholds. As reviewed by VanRullen and Koch (2003, p. 210), slow alpha waves ($\sim$ 10 Hz) seem to travel “more globally” (intermodal scope), and fast gamma waves ($\sim$ 40 Hz) “more locally” (intramodal scope). The frequency of alpha (gamma) waves might thus determine the central threshold for intermodal (intramodal) stimulus pairs (see also Kelber & Ulrich, 2025b). Indeed, their characteristic oscillatory frequencies agree remarkably well with the mean non-parametric threshold estimates obtained in Experiment 1 for intermodal (100 ms) and intramodal stimuli (25 ms). Neural oscillations may also be influenced by stimulus intensity (e.g., Schadow et al. 2007), which could potentially account for the less pronounced yet reliable threshold-invariance violations in Experiment 2. Overall, this multiple-oscillations account seems promising, but it will require further elaboration and formalization.

In summary, the present study tested the core assumption of independent-channels models that the threshold in a central timing mechanism does not depend on the latencies in the peripheral channels. Both non-parametric and parametric estimates of the central threshold were significantly higher for intermodal versus intramodal stimuli (Experiment 1), and also for weak versus strong intermodal stimuli (Experiment 2). These observations provide converging evidence against threshold invariance and thus challenge the dominant class of theoretical models of temporal-order and simultaneity perception.

## Supplementary Information

Below is the link to the electronic supplementary material.

Supplementary file 1 (pdf 701 KB)

## Data Availability

Raw data and experimental scripts are available at https://osf.io/yzpdu/.

## Code Availability

Analysis scripts are also available at https://osf.io/yzpdu/.

## Declarations

### Open Practices Statements

Both experiments were preregistered (Experiment 1: https://osf.io/u3dvm, Experiment 2: https://osf.io/eh3ms). Data, materials, and code are available (see above).
