# Supplementary material for: Evidence against independence of peripheral latencies and central threshold in temporal-order perception
Source: Psychon Bull Rev. 2026 Feb 17;33(3):62. doi: 10.3758/s13423-025-02797-0 (PMC12913284; doi:10.3758/s13423-025-02797-0)

Supplementary material for:

Evidence against independence of peripheral latencies and central threshold in temporal-order perception

Paul Kelber and Rolf Ulrich
University of Tübingen

Contents

Tabular representation of the observed psychometric functions **3**
Representation by response probability . . . 3
Experiment 1 . . . 3
Experiment 2 . . . 8
Representation by response frequency . . . 10
Experiment 1 . . . 10
Experiment 2 . . . 15

Tabular representation of the proportions of corrected/excluded responses **17**
Experiment 1 . . . 17
Experiment 2 . . . 17

Graphical representation of the two-threshold model fits **18**
Experiment 1 . . . 18
Experiment 2 . . . 22

Tabular representation of the observed psychometric functions

Representation by response probability

Experiment 1

Table S1 contains the observed responses probabilities in Experiment 1 pooled across the 20 sessions for each subject. All probabilities were rounded to three decimal places.

Table S1

Response probabilities in Experiment 1 (pooled across all sessions)

Subject	Stimuli	Response	Stimulus-onset difference d [ms]																								
			−200	−183	−167	−150	−133	−117	−100	−83	−67	−50	−33	−17	0	17	33	50	67	83	100	117	133	150	167	183	200
AN	Intramodal	R_{tb}	.000	.000	.000	.003	.003	.005	.003	.008	.005	.005	.021	.045	.177	.571	.880	.979	.990	1.000	.990	1.000	.990	.997	.992	.997	1.000
		R_{si}	.000	.000	.000	.000	.000	.003	.000	.000	.003	.018	.106	.355	.646	.409	.112	.015	.000	.000	.003	.000	.003	.000	.003	.000	.000
		R_{bt}	1.000	1.000	1.000	.997	.997	.992	.997	.992	.992	.977	.873	.599	.177	.020	.008	.005	.010	.000	.008	.000	.008	.003	.005	.003	.000
	Intermodal	R_{va}	.003	.015	.008	.008	.020	.013	.008	.015	.013	.005	.005	.013	.010	.025	.033	.040	.050	.065	.101	.139	.252	.347	.432	.548	.623
		R_{si}	.135	.189	.268	.369	.500	.614	.751	.773	.882	.932	.937	.947	.941	.947	.947	.932	.932	.907	.847	.826	.705	.593	.503	.381	.289
		R_{av}	.863	.795	.724	.624	.480	.374	.241	.212	.106	.063	.058	.040	.048	.028	.020	.028	.018	.028	.053	.035	.043	.060	.066	.071	.089
MS	Intramodal	R_{tb}	.000	.003	.005	.000	.003	.003	.003	.000	.000	.008	.013	.055	.136	.270	.666	.911	.970	.990	.985	.972	.992	.992	.990	.995	.990
		R_{si}	.000	.003	.005	.000	.000	.000	.008	.003	.008	.035	.191	.579	.730	.674	.317	.086	.025	.005	.013	.018	.003	.003	.008	.003	.010
		R_{bt}	1.000	.995	.990	1.000	.997	.997	.990	.997	.992	.957	.796	.365	.134	.056	.018	.003	.005	.005	.003	.010	.005	.005	.003	.003	.000
	Intermodal	R_{va}	.000	.005	.008	.013	.016	.023	.008	.036	.023	.043	.036	.023	.034	.067	.068	.094	.133	.210	.263	.372	.470	.573	.690	.783	.795
		R_{si}	.250	.311	.393	.419	.499	.575	.641	.696	.744	.795	.812	.858	.847	.842	.851	.834	.801	.749	.676	.579	.485	.384	.282	.184	.174
		R_{av}	.750	.684	.599	.569	.486	.402	.351	.268	.233	.161	.151	.119	.118	.090	.081	.071	.066	.041	.061	.048	.046	.043	.028	.033	.031
KS	Intramodal	R_{tb}	.000	.000	.000	.003	.000	.000	.000	.000	.000	.000	.010	.013	.020	.076	.243	.669	.905	.977	1.000	1.000	1.000	1.000	1.000	1.000	1.000
		R_{si}	.000	.000	.000	.000	.003	.000	.003	.003	.036	.094	.500	.804	.911	.865	.744	.320	.092	.023	.000	.000	.000	.000	.000	.000	.000
		R_{bt}	1.000	1.000	1.000	.997	.997	1.000	.997	.997	.964	.906	.490	.184	.069	.059	.013	.010	.003	.000	.000	.000	.000	.000	.000	.000	.000
	Intermodal	R_{va}	.029	.003	.016	.026	.043	.074	.064	.111	.129	.141	.125	.167	.136	.163	.211	.221	.294	.363	.403	.493	.573	.629	.684	.763	.737
		R_{si}	.109	.151	.203	.310	.380	.483	.609	.686	.729	.798	.838	.822	.851	.832	.778	.755	.696	.621	.589	.493	.419	.350	.305	.216	.255
		R_{av}	.862	.846	.781	.664	.578	.443	.327	.203	.142	.061	.037	.010	.013	.005	.010	.024	.010	.015	.008	.013	.008	.021	.011	.021	.008
WF	Intramodal	R_{tb}	.000	.000	.000	.003	.000	.000	.000	.000	.003	.000	.028	.040	.091	.229	.558	.864	.987	.995	.997	.997	1.000	1.000	1.000	1.000	1.000
		R_{si}	.000	.000	.000	.000	.000	.000	.003	.000	.003	.063	.247	.485	.662	.635	.347	.093	.005	.005	.003	.003	.000	.000	.000	.000	.000
		R_{bt}	1.000	1.000	1.000	.997	1.000	1.000	.997	1.000	.995	.937	.725	.475	.247	.136	.095	.043	.008	.000	.000	.000	.000	.000	.000	.000	.000
	Intermodal	R_{va}	.005	.000	.000	.010	.010	.008	.023	.038	.056	.073	.114	.129	.152	.227	.268	.346	.415	.492	.607	.715	.801	.874	.891	.954	.952
		R_{si}	.058	.071	.093	.139	.148	.131	.267	.313	.338	.407	.470	.518	.613	.584	.582	.567	.510	.482	.368	.265	.177	.108	.096	.036	.033
		R_{av}	.937	.929	.907	.851	.842	.861	.710	.649	.606	.520	.416	.354	.235	.189	.149	.087	.075	.025	.025	.020	.023	.018	.013	.010	.015
PK	Intramodal	R_{tb}	.000	.000	.003	.000	.000	.003	.000	.003	.000	.000	.008	.005	.008	.089	.486	.910	.997	1.000	1.000	1.000	1.000	.997	1.000	1.000	1.000
		R_{si}	.000	.000	.000	.000	.000	.000	.000	.000	.003	.033	.224	.693	.945	.896	.501	.088	.003	.000	.000	.000	.000	.000	.000	.000	.000
		R_{bt}	1.000	1.000	.997	1.000	1.000	.997	1.000	.997	.997	.967	.768	.302	.048	.015	.013	.003	.000	.000	.000	.000	.000	.003	.000	.000	.000
	Intermodal	R_{va}	.010	.010	.025	.010	.033	.048	.058	.068	.098	.111	.113	.118	.169	.212	.268	.330	.521	.722	.776	.899	.952	.972	.982	.982	.995
		R_{si}	.005	.018	.015	.030	.073	.116	.151	.280	.397	.572	.666	.771	.773	.733	.697	.640	.461	.260	.209	.095	.048	.025	.018	.010	.003
		R_{av}	.985	.972	.960	.960	.894	.837	.791	.652	.505	.317	.221	.111	.058	.055	.035	.030	.018	.018	.015	.005	.000	.003	.000	.008	.003

Note. See Table S8 for the corresponding response frequencies.

Tables S2–6 contain the observed responses probabilities divided into five practice levels (sessions 1–5, 6–10, 11–15, 16–20) for the subjects AN (Table S2), MS (Table S3), KS (Table S4), WF (Table S5), and PK (Table S6).

Table S2

Response probabilities of subject AN in Experiment 1 as a function of practice

Sessions	Stimuli	Response	Stimulus-onset difference d [ms]																							
			−200	−183	−167	−150	−133	−117	−100	−83	−67	−50	−33	−17	0	17	33	50	67	83	100	117	133	150	167	183	200
1–5	Intramodal	R_{tb}	.000	.000	.000	.000	.000	.010	.000	.000	.000	.010	.010	.010	.144	.402	.884	.969	1.000	1.000	1.000	1.000	1.000	1.000	1.000	1.000	1.000
		R_{si}	.000	.000	.000	.000	.000	.000	.000	.000	.000	.031	.124	.316	.608	.567	.105	.031	.000	.000	.000	.000	.000	.000	.000	.000	.000
		R_{bt}	1.000	1.000	1.000	1.000	1.000	.990	1.000	1.000	1.000	.959	.866	.673	.247	.031	.011	.000	.000	.000	.000	.000	.000	.000	.000	.000	.000
	Intermodal	R_{va}	.010	.051	.010	.020	.031	.041	.031	.051	.010	.020	.020	.051	.041	.092	.124	.153	.173	.214	.265	.306	.439	.500	.531	.567	.561
		R_{si}	.073	.143	.163	.214	.347	.361	.643	.561	.714	.806	.796	.796	.796	.816	.794	.755	.755	.684	.551	.582	.429	.347	.367	.289	.276
		R_{av}	.917	.806	.827	.765	.622	.598	.327	.388	.276	.173	.184	.153	.163	.092	.082	.092	.071	.102	.184	.112	.133	.153	.102	.144	.163
6–10	Intramodal	R_{tb}	.000	.000	.000	.000	.010	.000	.000	.000	.000	.010	.010	.030	.131	.480	.810	.980	.989	1.000	.980	1.000	.979	1.000	.990	1.000	1.000
		R_{si}	.000	.000	.000	.000	.000	.000	.000	.000	.010	.021	.083	.374	.727	.500	.190	.020	.000	.000	.010	.000	.011	.000	.010	.000	.000
		R_{bt}	1.000	1.000	1.000	1.000	.990	1.000	1.000	1.000	.990	.969	.906	.596	.141	.020	.000	.000	.011	.000	.010	.000	.011	.000	.000	.000	.000
	Intermodal	R_{va}	.000	.010	.000	.010	.040	.000	.000	.010	.030	.000	.000	.000	.000	.010	.000	.000	.020	.040	.060	.070	.313	.310	.440	.610	.745
		R_{si}	.020	.040	.081	.220	.253	.490	.670	.737	.890	.950	.950	1.000	.980	.980	1.000	1.000	.980	.960	.920	.920	.657	.630	.470	.300	.163
		R_{av}	.980	.949	.919	.770	.707	.510	.330	.253	.080	.050	.050	.000	.020	.010	.000	.000	.000	.000	.020	.010	.030	.060	.090	.090	.092
11–15	Intramodal	R_{tb}	.000	.000	.000	.000	.000	.010	.000	.000	.000	.000	.041	.050	.170	.643	.879	.969	.990	1.000	.980	1.000	1.000	.990	.990	1.000	1.000
		R_{si}	.000	.000	.000	.000	.000	.000	.000	.000	.000	.020	.144	.360	.690	.357	.121	.010	.000	.000	.000	.000	.000	.000	.000	.000	.000
		R_{bt}	1.000	1.000	1.000	1.000	1.000	.990	1.000	1.000	1.000	.980	.814	.590	.140	.000	.000	.021	.010	.000	.020	.000	.000	.010	.010	.000	.000
	Intermodal	R_{va}	.000	.000	.010	.000	.000	.010	.000	.000	.010	.000	.000	.000	.000	.000	.010	.010	.010	.010	.060	.111	.180	.420	.465	.667	.667
		R_{si}	.152	.160	.263	.388	.596	.730	.770	.860	.940	.980	1.000	.990	1.000	.990	.990	.970	.990	.980	.930	.869	.810	.550	.485	.313	.242
		R_{av}	.848	.840	.727	.612	.404	.260	.230	.140	.050	.020	.000	.010	.000	.010	.000	.020	.000	.010	.010	.020	.010	.030	.051	.020	.091
16–20	Intramodal	R_{tb}	.000	.000	.000	.010	.000	.000	.010	.030	.020	.000	.021	.090	.263	.758	.949	1.000	.979	1.000	1.000	1.000	.980	1.000	.990	.990	1.000
		R_{si}	.000	.000	.000	.000	.000	.010	.000	.000	.000	.000	.072	.370	.556	.212	.030	.000	.000	.000	.000	.000	.000	.000	.000	.000	.000
		R_{bt}	.000	.000	.000	.010	.000	.000	.010	.030	.020	.000	.021	.090	.263	.758	.949	1.000	.979	1.000	1.000	1.000	.980	1.000	.990	.990	1.000
	Intermodal	R_{va}	.000	.000	.010	.000	.010	.000	.000	.000	.000	.000	.000	.000	.000	.000	.000	.000	.000	.000	.020	.070	.080	.160	.293	.350	.520
		R_{si}	.293	.414	.566	.650	.800	.869	.920	.929	.980	.990	1.000	1.000	.990	1.000	1.000	1.000	1.000	1.000	.980	.930	.920	.840	.687	.620	.470
		R_{av}	.707	.586	.424	.350	.190	.131	.080	.071	.020	.010	.000	.000	.010	.000	.000	.000	.000	.000	.000	.000	.000	.000	.020	.030	.010

Note. See Table S9 for the corresponding response frequencies.

Table S3

Response probabilities of subject MS in Experiment 1 as a function of practice

Sessions	Stimuli	Response	Stimulus-onset difference d [ms]																								
			−200	−183	−167	−150	−133	−117	−100	−83	−67	−50	−33	−17	0	17	33	50	67	83	100	117	133	150	167	183	200
1–5	Intramodal	R_{tb}	.000	.000	.010	.000	.000	.000	.000	.000	.000	.010	.031	.124	.316	.469	.755	.908	.990	.990	1.000	.969	1.000	1.000	.990	1.000	.990
		R_{si}	.000	.000	.000	.000	.000	.000	.010	.000	.010	.041	.122	.309	.429	.427	.204	.082	.000	.010	.000	.021	.000	.000	.010	.000	.010
		R_{bt}	1.000	1.000	.990	1.000	1.000	1.000	.990	1.000	.990	.948	.847	.567	.255	.104	.041	.010	.010	.000	.000	.010	.000	.000	.000	.000	.000
	Intermodal	R_{va}	.000	.021	.021	.031	.021	.041	.020	.082	.051	.133	.093	.062	.117	.194	.175	.240	.320	.430	.505	.571	.643	.635	.804	.773	.792
		R_{si}	.122	.134	.144	.113	.292	.235	.296	.278	.367	.408	.474	.542	.468	.452	.557	.490	.454	.409	.299	.245	.214	.229	.103	.113	.083
		R_{av}	.878	.845	.835	.856	.688	.724	.684	.639	.582	.459	.433	.396	.415	.355	.268	.271	.227	.161	.196	.184	.143	.135	.093	.113	.125
6–10	Intramodal	R_{tb}	.000	.000	.010	.000	.000	.000	.000	.000	.000	.010	.010	.010	.030	.194	.590	.929	.980	.990	.990	.990	1.000	.980	.990	1.000	.980
		R_{si}	.000	.000	.000	.000	.000	.000	.000	.000	.000	.060	.263	.690	.929	.786	.400	.071	.010	.000	.010	.000	.000	.010	.000	.000	.020
		R_{bt}	1.000	1.000	.990	1.000	1.000	1.000	1.000	1.000	1.000	.930	.727	.300	.040	.020	.010	.000	.010	.010	.000	.010	.000	.010	.010	.000	.000
	Intermodal	R_{va}	.000	.000	.000	.000	.010	.010	.010	.030	.010	.000	.020	.020	.000	.031	.020	.120	.102	.265	.280	.398	.510	.735	.747	.918	.940
		R_{si}	.333	.434	.490	.535	.643	.717	.677	.798	.810	.900	.867	.939	.949	.958	.940	.860	.888	.735	.700	.602	.470	.235	.253	.082	.060
		R_{av}	.667	.566	.510	.465	.347	.273	.313	.172	.180	.100	.112	.040	.051	.010	.040	.020	.010	.000	.020	.000	.020	.031	.000	.000	.000
11–15	Intramodal	R_{tb}	.000	.010	.000	.000	.000	.010	.000	.000	.000	.000	.010	.070	.160	.232	.680	.940	.950	.990	.980	1.000	1.000	.990	.990	1.000	1.000
		R_{si}	.000	.000	.000	.000	.000	.000	.020	.010	.020	.020	.170	.640	.730	.717	.320	.060	.050	.000	.010	.000	.000	.000	.010	.000	.000
		R_{bt}	1.000	.990	1.000	1.000	1.000	.990	.980	.990	.980	.980	.820	.290	.110	.051	.000	.000	.000	.010	.010	.000	.000	.010	.000	.000	.000
	Intermodal	R_{va}	.000	.000	.010	.010	.011	.021	.000	.010	.010	.021	.010	.000	.000	.030	.010	.000	.031	.040	.081	.194	.323	.440	.596	.727	.643
		R_{si}	.261	.361	.515	.535	.547	.691	.812	.835	.939	.957	.979	.989	.990	.970	.980	1.000	.959	.960	.899	.806	.677	.560	.404	.273	.357
		R_{av}	.739	.639	.475	.455	.442	.289	.188	.155	.051	.021	.010	.011	.010	.000	.010	.000	.010	.000	.020	.000	.000	.000	.000	.000	.000
16–20	Intramodal	R_{tb}	.000	.000	.000	.000	.010	.000	.010	.000	.000	.010	.000	.020	.040	.190	.640	.869	.959	.990	.970	.930	.970	1.000	.990	.980	.990
		R_{si}	.000	.010	.020	.000	.000	.000	.000	.000	.000	.020	.210	.670	.830	.760	.340	.131	.041	.010	.030	.050	.010	.000	.010	.010	.010
		R_{bt}	1.000	.990	.980	1.000	.990	1.000	.990	1.000	1.000	.970	.790	.310	.130	.050	.020	.000	.000	.000	.000	.020	.020	.000	.000	.010	.000
	Intermodal	R_{va}	.000	.000	.000	.010	.021	.021	.000	.020	.021	.020	.022	.010	.022	.020	.071	.021	.081	.120	.188	.327	.402	.485	.611	.717	.802
		R_{si}	.287	.312	.419	.485	.510	.660	.784	.869	.862	.919	.935	.958	.978	.970	.919	.979	.899	.870	.802	.663	.577	.505	.368	.263	.198
		R_{av}	.713	.688	.581	.505	.469	.320	.216	.111	.117	.061	.043	.031	.000	.010	.010	.000	.020	.010	.010	.010	.021	.010	.021	.020	.000

Note. See Table S10 for the corresponding response frequencies.

Table S4

Response probabilities of subject KS in Experiment 1 as a function of practice

Sessions	Stimuli	Response	Stimulus-onset difference d [ms]																								
			−200	−183	−167	−150	−133	−117	−100	−83	−67	−50	−33	−17	0	17	33	50	67	83	100	117	133	150	167	183	200
1–5	Intramodal	R_{tb}	.000	.000	.000	.000	.000	.000	.000	.000	.000	.000	.021	.041	.084	.177	.419	.708	.907	.980	1.000	1.000	1.000	1.000	1.000	1.000	1.000
		R_{si}	.000	.000	.000	.000	.000	.000	.010	.010	.020	.051	.229	.515	.663	.604	.538	.250	.082	.020	.000	.000	.000	.000	.000	.000	.000
		R_{bt}	1.000	1.000	1.000	1.000	1.000	1.000	.990	.990	.980	.949	.750	.443	.253	.219	.043	.042	.010	.000	.000	.000	.000	.000	.000	.000	.000
	Intermodal	R_{va}	.075	.000	.054	.043	.115	.085	.052	.183	.175	.198	.135	.223	.219	.221	.274	.242	.385	.438	.438	.438	.547	.585	.702	.684	.740
		R_{si}	.183	.234	.161	.394	.354	.511	.604	.645	.691	.719	.760	.755	.771	.758	.716	.703	.594	.521	.531	.510	.442	.372	.266	.276	.260
		R_{av}	.742	.766	.785	.564	.531	.404	.344	.172	.134	.083	.104	.021	.010	.021	.011	.055	.021	.042	.031	.052	.011	.043	.032	.041	.000
6–10	Intramodal	R_{tb}	.000	.000	.000	.000	.000	.000	.000	.000	.000	.000	.010	.000	.000	.030	.186	.505	.905	.970	1.000	1.000	1.000	1.000	1.000	1.000	1.000
		R_{si}	.000	.000	.000	.000	.010	.000	.000	.000	.082	.165	.670	.930	.990	.960	.814	.495	.095	.030	.000	.000	.000	.000	.000	.000	.000
		R_{bt}	1.000	1.000	1.000	1.000	.990	1.000	1.000	1.000	.918	.835	.320	.070	.010	.010	.000	.000	.000	.000	.000	.000	.000	.000	.000	.000	.000
	Intermodal	R_{va}	.020	.010	.010	.063	.022	.165	.074	.143	.138	.116	.146	.214	.129	.146	.235	.271	.286	.333	.444	.554	.557	.656	.653	.707	.714
		R_{si}	.061	.134	.153	.253	.407	.443	.649	.673	.755	.811	.844	.776	.860	.854	.745	.698	.704	.646	.556	.446	.423	.312	.337	.263	.276
		R_{av}	.919	.856	.837	.684	.571	.392	.277	.184	.106	.074	.010	.010	.011	.000	.020	.031	.010	.020	.000	.000	.021	.031	.011	.030	.010
11–15	Intramodal	R_{tb}	.000	.000	.000	.010	.000	.000	.000	.000	.000	.000	.010	.000	.000	.061	.196	.724	.888	.970	1.000	1.000	1.000	1.000	1.000	1.000	1.000
		R_{si}	.000	.000	.000	.000	.000	.000	.000	.000	.031	.100	.592	.919	1.000	.939	.794	.276	.112	.030	.000	.000	.000	.000	.000	.000	.000
		R_{bt}	1.000	1.000	1.000	.990	1.000	1.000	1.000	1.000	.969	.900	.398	.081	.000	.000	.010	.000	.000	.000	.000	.000	.000	.000	.000	.000	.000
	Intermodal	R_{va}	.000	.000	.000	.000	.032	.032	.083	.094	.185	.193	.135	.152	.147	.186	.188	.240	.229	.371	.424	.505	.500	.635	.624	.787	.656
		R_{si}	.101	.104	.276	.323	.389	.500	.562	.656	.652	.773	.844	.848	.832	.814	.802	.750	.771	.629	.576	.495	.500	.365	.376	.202	.333
		R_{av}	.899	.896	.724	.677	.579	.468	.354	.250	.163	.034	.021	.000	.021	.000	.010	.010	.000	.000	.000	.000	.000	.000	.000	.011	.010
16–20	Intramodal	R_{tb}	.000	.000	.000	.000	.000	.000	.000	.000	.000	.000	.000	.010	.000	.040	.180	.735	.920	.990	1.000	1.000	1.000	1.000	1.000	1.000	1.000
		R_{si}	.000	.000	.000	.000	.000	.000	.000	.000	.010	.062	.505	.844	.980	.950	.820	.265	.080	.010	.000	.000	.000	.000	.000	.000	.000
		R_{bt}	1.000	1.000	1.000	1.000	1.000	1.000	1.000	1.000	.990	.938	.495	.146	.020	.010	.000	.000	.000	.000	.000	.000	.000	.000	.000	.000	.000
	Intermodal	R_{va}	.021	.000	.000	.000	.000	.011	.044	.022	.021	.061	.084	.081	.051	.101	.152	.134	.276	.312	.306	.480	.688	.638	.755	.878	.837
		R_{si}	.096	.134	.221	.271	.370	.478	.622	.772	.814	.888	.905	.909	.939	.899	.848	.866	.714	.688	.694	.520	.312	.351	.245	.122	.153
		R_{av}	.883	.866	.779	.729	.630	.511	.333	.207	.165	.051	.011	.010	.010	.000	.000	.000	.010	.000	.000	.000	.000	.011	.000	.000	.010

Note. See Table S11 for the corresponding response frequencies.

Table S5

Response probabilities of subject WF in Experiment 1 as a function of practice

Sessions	Stimuli	Response	Stimulus-onset difference d [ms]																								
			−200	−183	−167	−150	−133	−117	−100	−83	−67	−50	−33	−17	0	17	33	50	67	83	100	117	133	150	167	183	200
1–5	Intramodal	R_{tb}	.000	.000	.000	.000	.000	.000	.000	.000	.010	.000	.092	.122	.153	.378	.653	.929	.990	.990	.990	.990	1.000	1.000	1.000	1.000	1.000
		R_{si}	.000	.000	.000	.000	.000	.000	.010	.000	.000	.102	.388	.622	.673	.561	.296	.061	.000	.010	.010	.010	.000	.000	.000	.000	.000
		R_{bt}	1.000	1.000	1.000	1.000	1.000	1.000	.990	1.000	.990	.898	.520	.255	.173	.061	.051	.010	.010	.000	.000	.000	.000	.000	.000	.000	.000
	Intermodal	R_{va}	.000	.000	.000	.041	.031	.010	.031	.061	.041	.041	.072	.061	.112	.163	.173	.237	.286	.378	.551	.633	.776	.857	.835	.907	.939
		R_{si}	.093	.153	.224	.186	.344	.255	.480	.429	.474	.598	.619	.653	.673	.633	.673	.608	.612	.551	.408	.306	.173	.102	.134	.052	.031
		R_{av}	.907	.847	.776	.773	.625	.735	.490	.510	.485	.361	.309	.286	.214	.204	.153	.155	.102	.071	.041	.061	.051	.041	.031	.041	.031
6–10	Intramodal	R_{tb}	.000	.000	.000	.010	.000	.000	.000	.000	.000	.000	.000	.000	.020	.050	.380	.740	.960	.990	1.000	1.000	1.000	1.000	1.000	1.000	1.000
		R_{si}	.000	.000	.000	.000	.000	.000	.000	.000	.010	.140	.500	.810	.949	.930	.590	.260	.020	.010	.000	.000	.000	.000	.000	.000	.000
		R_{bt}	1.000	1.000	1.000	.990	1.000	1.000	1.000	1.000	.990	.860	.500	.190	.030	.020	.030	.000	.020	.000	.000	.000	.000	.000	.000	.000	.000
	Intermodal	R_{va}	.010	.000	.000	.000	.010	.000	.000	.010	.000	.010	.040	.000	.020	.030	.000	.140	.160	.230	.410	.660	.720	.860	.850	.960	.960
		R_{si}	.071	.090	.100	.242	.182	.180	.343	.450	.460	.470	.500	.740	.800	.810	.830	.820	.820	.760	.570	.340	.280	.130	.150	.040	.030
		R_{av}	.919	.910	.900	.758	.808	.820	.657	.540	.540	.520	.460	.260	.180	.160	.170	.040	.020	.010	.020	.000	.000	.010	.000	.000	.010
11–15	Intramodal	R_{tb}	.000	.000	.000	.000	.000	.000	.000	.000	.000	.000	.010	.010	.071	.212	.520	.870	1.000	1.000	1.000	1.000	1.000	1.000	1.000	1.000	1.000
		R_{si}	.000	.000	.000	.000	.000	.000	.000	.000	.000	.010	.091	.420	.778	.707	.380	.050	.000	.000	.000	.000	.000	.000	.000	.000	.000
		R_{bt}	1.000	1.000	1.000	1.000	1.000	1.000	1.000	1.000	1.000	.990	.899	.570	.152	.081	.100	.080	.000	.000	.000	.000	.000	.000	.000	.000	.000
	Intermodal	R_{va}	.000	.000	.000	.000	.000	.000	.010	.010	.031	.071	.082	.110	.163	.130	.280	.360	.460	.530	.582	.636	.760	.800	.890	.950	.930
		R_{si}	.071	.040	.050	.130	.070	.090	.230	.340	.392	.485	.622	.530	.622	.680	.620	.570	.440	.470	.408	.354	.240	.190	.100	.050	.070
		R_{av}	.929	.960	.950	.870	.930	.910	.760	.650	.577	.444	.296	.360	.214	.190	.100	.070	.100	.000	.010	.010	.000	.010	.010	.000	.000
16–20	Intramodal	R_{tb}	.000	.000	.000	.000	.000	.000	.000	.000	.000	.000	.010	.031	.120	.280	.680	.919	1.000	1.000	1.000	1.000	1.000	1.000	1.000	1.000	1.000
		R_{si}	.000	.000	.000	.000	.000	.000	.000	.000	.000	.000	.010	.082	.250	.340	.120	.000	.000	.000	.000	.000	.000	.000	.000	.000	.000
		R_{bt}	1.000	1.000	1.000	1.000	1.000	1.000	1.000	1.000	1.000	1.000	.980	.888	.630	.380	.200	.081	.000	.000	.000	.000	.000	.000	.000	.000	.000
	Intermodal	R_{va}	.010	.000	.000	.000	.000	.020	.050	.071	.152	.170	.263	.347	.313	.586	.629	.656	.750	.830	.888	.929	.949	.980	.990	1.000	.980
		R_{si}	.000	.000	.000	.000	.000	.000	.020	.031	.030	.080	.141	.143	.354	.212	.196	.260	.170	.150	.082	.061	.010	.010	.000	.000	.000
		R_{av}	.990	1.000	1.000	1.000	1.000	.980	.930	.898	.818	.750	.596	.510	.333	.202	.175	.083	.080	.020	.031	.010	.041	.010	.010	.000	.020

Note. See Table S12 for the corresponding response frequencies.

Table S6

Response probabilities of subject PK in Experiment 1 as a function of practice

Sessions	Stimuli	Response	Stimulus-onset difference d [ms]																						
			−200	−183	−167	−150	−133	−117	−100	−83	−67	−50	−33	−17	0	17	33	50	67	83	100	117	133	150	167	183	200
1–5	Intramodal	R_{tb}	.000	.000	.000	.000	.000	.000	.000	.010	.000	.000	.020	.010	.000	.102	.433	.827	1.000	1.000	1.000	1.000	1.000	1.000	1.000	1.000	1.000
		R_{si}	.000	.000	.000	.000	.000	.000	.000	.000	.010	.102	.429	.857	.969	.888	.515	.163	.000	.000	.000	.000	.000	.000	.000	.000	.000
		R_{bt}	1.000	1.000	1.000	1.000	1.000	1.000	1.000	.990	.990	.898	.551	.133	.031	.010	.052	.010	.000	.000	.000	.000	.000	.000	.000	.000	.000
	Intermodal	R_{va}	.031	.031	.041	.021	.092	.122	.144	.134	.173	.124	.092	.112	.214	.286	.240	.337	.469	.633	.786	.837	.939	.979	.969	.959	.990
		R_{si}	.010	.020	.010	.031	.082	.092	.144	.309	.367	.649	.755	.796	.724	.663	.719	.642	.490	.337	.173	.143	.061	.021	.031	.031	.000
		R_{av}	.959	.949	.949	.948	.827	.786	.711	.557	.459	.227	.153	.092	.061	.051	.042	.021	.041	.031	.041	.020	.000	.000	.000	.010	.010
6–10	Intramodal	R_{tb}	.000	.000	.010	.000	.000	.000	.000	.000	.000	.000	.000	.000	.020	.081	.460	.900	1.000	1.000	1.000	1.000	1.000	1.000	1.000	1.000	1.000
		R_{si}	.000	.000	.000	.000	.000	.000	.000	.000	.000	.030	.200	.660	.910	.909	.540	.100	.000	.000	.000	.000	.000	.000	.000	.000	.000
		R_{bt}	1.000	1.000	.990	1.000	1.000	1.000	1.000	1.000	1.000	.970	.800	.340	.070	.010	.000	.000	.000	.000	.000	.000	.000	.000	.000	.000	.000
	Intermodal	R_{va}	.010	.000	.050	.000	.040	.060	.060	.080	.180	.140	.210	.202	.232	.253	.390	.380	.620	.788	.750	.930	.980	.970	.990	.990	1.000
		R_{si}	.000	.000	.040	.050	.040	.170	.230	.320	.430	.570	.620	.727	.737	.707	.590	.600	.370	.202	.230	.070	.020	.030	.010	.000	.000
		R_{av}	.990	1.000	.910	.950	.920	.770	.710	.600	.390	.290	.170	.071	.030	.040	.020	.020	.010	.010	.020	.000	.000	.000	.000	.010	.000
11–15	Intramodal	R_{tb}	.000	.000	.000	.000	.000	.000	.000	.000	.000	.000	.000	.010	.000	.082	.530	.980	1.000	1.000	1.000	1.000	1.000	1.000	1.000	1.000	1.000
		R_{si}	.000	.000	.000	.000	.000	.000	.000	.000	.000	.000	.111	.570	.940	.878	.470	.020	.000	.000	.000	.000	.000	.000	.000	.000	.000
		R_{bt}	1.000	1.000	1.000	1.000	1.000	1.000	1.000	1.000	1.000	1.000	.889	.420	.060	.041	.000	.000	.000	.000	.000	.000	.000	.000	.000	.000	.000
	Intermodal	R_{va}	.000	.010	.000	.010	.000	.000	.010	.050	.030	.090	.120	.100	.090	.150	.270	.364	.520	.790	.800	.930	.960	.980	1.000	1.000	.990
		R_{si}	.010	.020	.000	.010	.070	.120	.140	.330	.470	.540	.630	.820	.830	.760	.710	.586	.470	.200	.200	.070	.040	.010	.000	.000	.010
		R_{av}	.990	.970	1.000	.980	.930	.880	.850	.620	.500	.370	.250	.080	.080	.090	.020	.051	.010	.010	.000	.000	.000	.010	.000	.000	.000
16–20	Intramodal	R_{tb}	.000	.000	.000	.000	.000	.010	.000	.000	.000	.000	.010	.000	.010	.090	.520	.930	.990	1.000	1.000	1.000	1.000	.990	1.000	1.000	1.000
		R_{si}	.000	.000	.000	.000	.000	.000	.000	.000	.000	.000	.160	.690	.960	.910	.480	.070	.010	.000	.000	.000	.000	.000	.000	.000	.000
		R_{bt}	1.000	1.000	1.000	1.000	1.000	.990	1.000	1.000	1.000	1.000	.830	.310	.030	.000	.000	.000	.000	.000	.000	.000	.000	.010	.000	.000	.000
	Intermodal	R_{va}	.000	.000	.010	.010	.000	.010	.020	.010	.010	.090	.030	.060	.140	.160	.170	.240	.475	.677	.768	.900	.929	.960	.970	.980	1.000
		R_{si}	.000	.030	.010	.030	.100	.080	.090	.162	.320	.530	.660	.740	.800	.800	.770	.730	.515	.303	.232	.100	.071	.040	.030	.010	.000
		R_{av}	1.000	.970	.980	.960	.900	.910	.890	.828	.670	.380	.310	.200	.060	.040	.060	.030	.010	.020	.000	.000	.000	.000	.000	.010	.000

Note. See Table S13 For the corresponding response frequencies.

Experiment 2

Table S7 on the next page contains the observed responses probabilities in Experiment 2 pooled across the five sessions for each subject.

Table S7

Response probabilities in Experiment 2

Subject	Intensity	Response	Stimulus-onset difference d [ms]														
			−350	−300	−250	−200	−150	−100	−50	0	50	100	150	200	250	300	350
ER	Strong	R_{va}	.006	.006	.000	.042	.071	.054	.125	.095	.120	.214	.500	.619	.714	.792	.845
		R_{si}	.048	.054	.137	.208	.351	.683	.655	.762	.759	.667	.363	.280	.167	.095	.095
		R_{av}	.946	.940	.863	.750	.577	.263	.220	.143	.120	.119	.137	.101	.119	.113	.060
	Weak	R_{va}	.054	.054	.060	.084	.125	.131	.101	.107	.131	.204	.304	.389	.512	.619	.687
		R_{si}	.133	.238	.293	.323	.435	.607	.685	.696	.732	.641	.542	.485	.351	.262	.247
		R_{av}	.813	.708	.647	.593	.440	.262	.214	.196	.137	.156	.155	.126	.137	.119	.066
LD	Strong	R_{va}	.000	.011	.006	.017	.000	.000	.017	.006	.028	.084	.371	.669	.809	.910	.989
		R_{si}	.023	.068	.186	.486	.685	.921	.955	.949	.944	.899	.624	.331	.185	.090	.011
		R_{av}	.977	.921	.808	.497	.315	.079	.028	.045	.028	.017	.006	.000	.006	.000	.000
	Weak	R_{va}	.017	.000	.017	.006	.011	.028	.051	.040	.084	.136	.303	.469	.753	.860	.955
		R_{si}	.164	.307	.429	.588	.761	.893	.915	.915	.899	.853	.685	.525	.242	.135	.039
		R_{av}	.819	.693	.554	.407	.227	.079	.034	.045	.017	.011	.011	.006	.006	.006	.006
VS	Strong	R_{va}	.000	.000	.000	.006	.000	.011	.051	.023	.205	.744	.932	.966	.994	.983	.983
		R_{si}	.000	.000	.000	.011	.029	.136	.653	.949	.756	.250	.062	.023	.000	.006	.017
		R_{av}	1.000	1.000	1.000	.983	.971	.852	.295	.028	.040	.006	.006	.011	.006	.011	.000
	Weak	R_{va}	.006	.000	.011	.011	.006	.046	.114	.159	.211	.665	.869	.983	.977	.994	1.000
		R_{si}	.000	.000	.000	.011	.046	.137	.526	.812	.771	.330	.097	.011	.023	.000	.000
		R_{av}	.994	1.000	.989	.977	.949	.817	.360	.028	.017	.006	.034	.006	.000	.006	.000
CL	Strong	R_{va}	.006	.006	.012	.024	.006	.006	.041	.052	.254	.611	.787	.890	.949	.954	.955
		R_{si}	.065	.076	.183	.444	.760	.942	.930	.924	.728	.353	.195	.092	.040	.040	.040
		R_{av}	.929	.919	.805	.533	.234	.053	.029	.023	.018	.036	.017	.017	.011	.006	.006
	Weak	R_{va}	.006	.006	.000	.031	.012	.018	.031	.090	.200	.494	.723	.838	.948	.941	.959
		R_{si}	.170	.356	.482	.730	.876	.941	.938	.892	.768	.494	.247	.150	.046	.053	.041
		R_{av}	.824	.637	.518	.239	.112	.041	.031	.018	.032	.013	.030	.012	.006	.006	.000
ML	Strong	R_{va}	.000	.011	.006	.017	.006	.023	.023	.057	.210	.494	.649	.730	.830	.886	.903
		R_{si}	.023	.017	.023	.108	.224	.247	.409	.528	.625	.386	.247	.202	.102	.080	.051
		R_{av}	.977	.971	.971	.875	.770	.730	.568	.415	.165	.119	.103	.067	.068	.034	.046
	Weak	R_{va}	.023	.017	.023	.017	.053	.052	.091	.165	.277	.425	.545	.609	.676	.797	.828
		R_{si}	.064	.080	.130	.142	.222	.333	.451	.557	.561	.460	.348	.310	.233	.145	.126
		R_{av}	.913	.902	.847	.841	.725	.615	.457	.278	.162	.115	.107	.080	.091	.058	.046
MM	Strong	R_{va}	.017	.039	.046	.040	.017	.023	.000	.000	.006	.040	.125	.209	.318	.397	.615
		R_{si}	.185	.247	.389	.589	.787	.887	.972	.966	.944	.938	.818	.661	.557	.420	.276
		R_{av}	.798	.713	.566	.371	.197	.090	.028	.034	.051	.023	.057	.130	.125	.184	.109
	Weak	R_{va}	.028	.029	.029	.028	.011	.017	.000	.011	.011	.023	.022	.073	.107	.171	.230
		R_{si}	.282	.451	.594	.763	.915	.927	.977	.978	.983	.966	.972	.910	.870	.806	.697
		R_{av}	.689	.520	.377	.209	.074	.056	.023	.011	.006	.011	.006	.017	.023	.023	.073
FL	Strong	R_{va}	.006	.000	.017	.028	.075	.081	.147	.210	.347	.511	.506	.608	.678	.712	.703
		R_{si}	.113	.192	.328	.469	.638	.763	.806	.727	.625	.443	.466	.358	.277	.271	.280
		R_{av}	.881	.808	.655	.503	.287	.156	.047	.062	.028	.046	.028	.034	.045	.017	.017
	Weak	R_{va}	.023	.046	.079	.097	.136	.097	.202	.264	.372	.318	.491	.437	.460	.417	.434
		R_{si}	.237	.354	.469	.648	.761	.857	.763	.718	.593	.647	.474	.552	.506	.577	.554
		R_{av}	.740	.600	.452	.256	.102	.046	.035	.017	.035	.035	.034	.011	.034	.006	.011
RZ	Strong	R_{va}	.000	.006	.011	.022	.017	.062	.084	.096	.118	.360	.742	.882	.983	.978	.955
		R_{si}	.023	.023	.084	.169	.337	.689	.882	.899	.871	.640	.253	.112	.011	.017	.039
		R_{av}	.977	.971	.904	.809	.646	.249	.034	.006	.011	.000	.006	.006	.006	.006	.006
	Weak	R_{va}	.000	.000	.000	.022	.045	.068	.084	.130	.169	.311	.612	.797	.899	.927	.949
		R_{si}	.022	.011	.056	.118	.220	.525	.843	.847	.826	.684	.376	.192	.090	.056	.039
		R_{av}	.978	.989	.944	.860	.734	.407	.073	.023	.006	.006	.011	.011	.011	.017	.011
LF	Strong	R_{va}	.057	.092	.064	.164	.217	.246	.345	.331	.293	.356	.379	.429	.460	.409	.570
		R_{si}	.172	.214	.283	.433	.537	.667	.632	.657	.701	.644	.609	.560	.529	.591	.419
		R_{av}	.770	.694	.653	.404	.246	.088	.023	.011	.006	.000	.011	.011	.011	.000	.012
	Weak	R_{va}	.011	.029	.006	.024	.036	.052	.057	.094	.098	.092	.092	.087	.126	.103	.108
		R_{si}	.454	.517	.540	.714	.778	.884	.892	.883	.890	.896	.896	.895	.862	.885	.886
		R_{av}	.534	.453	.455	.262	.186	.064	.051	.023	.012	.012	.012	.017	.011	.011	.006
SE	Strong	R_{va}	.011	.000	.011	.011	.006	.000	.006	.011	.045	.143	.309	.676	.847	.966	.977
		R_{si}	.017	.057	.136	.352	.691	.909	.989	.977	.955	.834	.646	.312	.148	.028	.017
		R_{av}	.971	.943	.852	.636	.303	.091	.006	.011	.000	.023	.046	.011	.006	.006	.006
	Weak	R_{va}	.000	.006	.006	.011	.011	.017	.034	.029	.011	.165	.434	.766	.898	.949	.971
		R_{si}	.006	.034	.091	.176	.511	.800	.926	.954	.966	.830	.554	.223	.085	.034	.023
		R_{av}	.994	.960	.903	.812	.477	.183	.040	.017	.023	.006	.011	.011	.017	.017	.006

Note. See Table S14 for the corresponding response frequencies.

Representation by response frequency

Experiment 1

Table S8 contains the observed responses frequencies in Experiment 1 pooled across the 20 sessions for each subject.

Table S8

Response frequencies in Experiment 1 (pooled across all sessions)

Subject	Stimuli	Response	Stimulus-onset difference d [ms]																								
			−200	−183	−167	−150	−133	−117	−100	−83	−67	−50	−33	−17	0	17	33	50	67	83	100	117	133	150	167	183	200
AN	Intramodal	R_{tb}	0	0	0	1	1	2	1	3	2	2	8	18	70	225	346	382	377	393	389	393	387	393	389	396	391
		R_{si}	0	0	0	0	0	1	0	0	1	7	41	141	255	161	44	6	0	0	1	0	1	0	1	0	0
		R_{bt}	395	396	394	392	394	389	391	392	392	383	338	238	70	8	3	2	4	0	3	0	3	1	2	1	0
	Intermodal	R_{va}	1	6	3	3	8	5	3	6	5	2	2	5	4	10	13	16	20	26	40	55	100	138	171	217	246
		R_{si}	53	75	106	146	198	243	299	306	350	369	372	375	370	375	376	369	371	360	337	328	280	236	199	151	114
		R_{av}	339	315	286	247	190	148	96	84	42	25	23	16	19	11	8	11	7	11	21	14	17	24	26	28	35
MS	Intramodal	R_{tb}	0	1	2	0	1	1	1	0	0	3	5	22	54	106	265	360	383	394	390	384	393	394	392	394	392
		R_{si}	0	1	2	0	0	0	3	1	3	14	76	230	290	265	126	34	10	2	5	7	1	1	3	1	4
		R_{bt}	396	396	394	398	394	395	388	395	392	379	316	145	53	22	7	1	2	2	1	4	2	2	1	1	0
	Intermodal	R_{va}	0	2	3	5	6	9	3	14	9	17	14	9	13	26	27	37	52	82	103	146	185	225	269	307	310
		R_{si}	94	120	153	165	192	225	250	273	290	311	312	331	322	326	336	327	314	293	265	227	191	151	110	72	68
		R_{av}	282	264	233	224	187	157	137	105	91	63	58	46	45	35	32	28	26	16	24	19	18	17	11	13	12
KS	Intramodal	R_{tb}	0	0	0	1	0	0	0	0	0	0	4	5	8	30	94	259	353	387	397	396	395	396	396	398	394
		R_{si}	0	0	0	0	1	0	1	1	14	37	195	315	359	340	288	124	36	9	0	0	0	0	0	0	0
		R_{bt}	396	396	395	397	395	398	397	394	378	355	191	72	27	23	5	4	1	0	0	0	0	0	0	0	0
	Intermodal	R_{va}	11	1	6	10	16	28	24	42	49	53	48	64	52	62	82	84	114	141	158	187	219	239	260	297	286
		R_{si}	42	58	78	117	142	182	229	260	277	301	321	315	325	316	302	287	270	241	231	187	160	133	116	84	99
		R_{av}	332	325	300	251	216	167	123	77	54	23	14	4	5	2	4	9	4	6	3	5	3	8	4	8	3
WF	Intramodal	R_{tb}	0	0	0	1	0	0	0	0	1	0	11	16	36	91	222	343	393	395	395	397	396	397	396	397	397
		R_{si}	0	0	0	0	0	0	1	0	1	25	98	192	262	252	138	37	2	2	1	1	0	0	0	0	0
		R_{bt}	395	395	395	396	394	397	394	394	396	373	288	188	98	54	38	17	3	0	0	0	0	0	0	0	0
	Intermodal	R_{va}	2	0	0	4	4	3	9	15	22	29	45	51	60	90	106	136	165	196	239	283	317	347	353	376	378
		R_{si}	23	28	37	55	58	52	106	124	133	161	185	205	242	232	230	223	203	192	145	105	70	43	38	14	13
		R_{av}	369	366	360	336	331	341	282	257	238	206	164	140	93	75	59	34	30	10	10	8	9	7	5	4	6
PK	Intramodal	R_{tb}	0	0	1	0	0	1	0	1	0	0	3	2	3	35	193	362	396	396	398	398	398	397	397	398	398
		R_{si}	0	0	0	0	0	0	0	0	1	13	89	276	375	354	199	35	1	0	0	0	0	0	0	0	0
		R_{bt}	397	398	397	398	398	397	398	397	397	385	305	120	19	6	5	1	0	0	0	0	0	1	0	0	0
	Intermodal	R_{va}	4	4	10	4	13	19	23	27	39	44	45	47	67	84	106	130	207	286	308	358	378	386	391	389	395
		R_{si}	2	7	6	12	29	46	60	111	158	227	265	306	307	291	276	252	183	103	83	38	19	10	7	4	1
		R_{av}	390	387	382	381	356	333	314	258	201	126	88	44	23	22	14	12	7	7	6	2	0	1	0	3	1

Note. See Table S1 for the corresponding response probabilities.

Tables S9–13 contain the observed responses frequencies divided into five practice levels (sessions 1–5, 6–10, 11–15, 16–20) for the subjects AN (Table S9), MS (Table S10), KS (Table S11), WF (Table S12), and PK (Table S13).

Table S9

Response frequencies of subject AN in Experiment 1 as a function of practice

Sessions	Stimuli	Response	Stimulus-onset difference d [ms]																								
			−200	−183	−167	−150	−133	−117	−100	−83	−67	−50	−33	−17	0	17	33	50	67	83	100	117	133	150	167	183	200
1–5	Intramodal	R_{tb}	0	0	0	0	0	1	0	0	0	1	1	1	14	39	84	93	98	96	96	98	98	97	97	97	97
		R_{si}	0	0	0	0	0	0	0	0	0	3	12	31	59	55	10	3	0	0	0	0	0	0	0	0	0
		R_{bt}	98	98	98	97	97	97	98	97	97	94	84	66	24	3	1	0	0	0	0	0	0	0	0	0	0
	Intermodal	R_{va}	1	5	1	2	3	4	3	5	1	2	2	5	4	9	12	15	17	21	26	30	43	49	52	55	55
		R_{si}	7	14	16	21	34	35	63	55	70	79	78	78	78	80	77	74	74	67	54	57	42	34	36	28	27
		R_{av}	88	79	81	75	61	58	32	38	27	17	18	15	16	9	8	9	7	10	18	11	13	15	10	14	16
6–10	Intramodal	R_{tb}	0	0	0	0	1	0	0	0	0	1	1	3	13	48	81	97	89	99	97	98	93	98	98	100	99
		R_{si}	0	0	0	0	0	0	0	0	1	2	8	37	72	50	19	2	0	0	1	0	1	0	1	0	0
		R_{bt}	98	99	99	98	98	97	96	98	99	94	87	59	14	2	0	0	1	0	1	0	1	0	0	0	0
	Intermodal	R_{va}	0	1	0	1	4	0	0	1	3	0	0	0	0	1	0	0	2	4	6	7	31	31	44	61	73
		R_{si}	2	4	8	22	25	49	67	73	89	95	95	100	98	97	100	99	98	95	92	92	65	63	47	30	16
		R_{av}	97	94	91	77	70	51	33	25	8	5	5	0	2	1	0	0	0	0	2	1	3	6	9	9	9
11–15	Intramodal	R_{tb}	0	0	0	0	0	1	0	0	0	0	4	5	17	63	87	94	96	98	97	98	99	98	97	100	98
		R_{si}	0	0	0	0	0	0	0	0	0	2	14	36	69	35	12	1	0	0	0	0	0	0	0	0	0
		R_{bt}	99	99	99	100	100	96	98	100	99	98	79	59	14	0	0	2	1	0	2	0	0	1	1	0	0
	Intermodal	R_{va}	0	0	1	0	0	1	0	0	1	0	0	0	0	0	1	1	1	1	6	11	18	42	46	66	66
		R_{si}	15	16	26	38	59	73	77	86	94	97	99	98	98	98	99	97	99	98	93	86	81	55	48	31	24
		R_{av}	84	84	72	60	40	26	23	14	5	2	0	1	0	1	0	2	0	1	1	2	1	3	5	2	9
16–20	Intramodal	R_{tb}	0	0	0	1	0	0	1	3	2	0	2	9	26	75	94	98	94	100	99	99	97	100	97	99	97
		R_{si}	0	0	0	0	0	1	0	0	0	0	7	37	55	21	3	0	0	0	0	0	0	0	0	0	0
		R_{bt}	100	100	98	97	99	99	99	97	97	97	88	54	18	3	2	0	2	0	0	0	2	0	1	1	0
	Intermodal	R_{va}	0	0	1	0	1	0	0	0	0	0	0	0	0	0	0	0	0	0	2	7	8	16	29	35	52
		R_{si}	29	41	56	65	80	86	92	92	97	98	100	99	96	100	100	99	100	100	98	93	92	84	68	62	47
		R_{av}	70	58	42	35	19	13	8	7	2	1	0	0	1	0	0	0	0	0	0	0	0	0	2	3	1

Note. See Table S2 for the corresponding response probabilities.

Table S10

Response frequencies of subject MS in Experiment 1 as a function of practice

Sessions	Stimuli	Response	Stimulus-onset difference d [ms]																							
			−200	−183	−167	−150	−133	−117	−100	−83	−67	−50	−33	−17	0	17	33	50	67	83	100	117	133	150	167	183	200
1–5	Intramodal	R_{tb}	0	0	1	0	0	0	0	0	0	1	3	12	31	45	74	89	97	97	97	94	97	97	97	97	97
		R_{si}	0	0	0	0	0	0	1	0	1	4	12	30	42	41	20	8	0	1	0	2	0	0	1	0	1
		R_{bt}	97	98	97	98	97	98	95	97	96	92	83	55	25	10	4	1	1	0	0	1	0	0	0	0	0
	Intermodal	R_{va}	0	2	2	3	2	4	2	8	5	13	9	6	11	18	17	23	31	40	49	56	63	61	78	75	76
		R_{si}	12	13	14	11	28	23	29	27	36	40	46	52	44	42	54	47	44	38	29	24	21	22	10	11	8
		R_{av}	86	82	81	83	66	71	67	62	57	45	42	38	39	33	26	26	22	15	19	18	14	13	9	11	12
6–10	Intramodal	R_{tb}	0	0	1	0	0	0	0	0	0	1	1	1	3	19	59	91	97	99	98	98	100	98	98	100	98
		R_{si}	0	0	0	0	0	0	0	0	0	6	26	69	92	77	40	7	1	0	1	0	0	1	0	0	2
		R_{bt}	99	100	99	100	100	99	97	100	99	93	72	30	4	2	1	0	1	1	0	1	0	1	1	0	0
	Intermodal	R_{va}	0	0	0	0	1	1	1	3	1	0	2	2	0	3	2	12	10	26	28	39	51	72	74	89	94
		R_{si}	33	43	49	53	63	71	67	79	81	90	85	93	94	92	94	86	87	72	70	59	47	23	25	8	6
		R_{av}	66	56	51	46	34	27	31	17	18	10	11	4	5	1	4	2	1	0	2	0	2	3	0	0	0
11–15	Intramodal	R_{tb}	0	1	0	0	0	1	0	0	0	0	1	7	16	23	68	94	95	99	98	99	100	99	99	100	98
		R_{si}	0	0	0	0	0	0	2	1	2	2	17	64	73	71	32	6	5	0	1	0	0	0	1	0	0
		R_{bt}	100	99	100	100	99	99	98	99	97	98	82	29	11	5	0	0	0	1	1	0	0	1	0	0	0
	Intermodal	R_{va}	0	0	1	1	1	2	0	1	1	2	1	0	0	3	1	0	3	4	8	19	32	44	59	72	63
		R_{si}	24	35	51	53	52	67	78	81	92	90	94	94	96	96	97	100	94	96	89	79	67	56	40	27	35
		R_{av}	68	62	47	45	42	28	18	15	5	2	1	1	1	0	1	0	1	0	2	0	0	0	0	0	0
16–20	Intramodal	R_{tb}	0	0	0	0	1	0	1	0	0	1	0	2	4	19	64	86	94	99	97	93	96	100	98	97	99
		R_{si}	0	1	2	0	0	0	0	0	0	2	21	67	83	76	34	13	4	1	3	5	1	0	1	1	1
		R_{bt}	100	99	98	100	98	99	98	99	100	96	79	31	13	5	2	0	0	0	0	2	2	0	0	1	0
	Intermodal	R_{va}	0	0	0	1	2	2	0	2	2	2	2	1	2	2	7	2	8	12	18	32	39	48	58	71	77
		R_{si}	25	29	39	48	49	64	76	86	81	91	87	92	88	96	91	94	89	87	77	65	56	50	35	26	19
		R_{av}	62	64	54	50	45	31	21	11	11	6	4	3	0	1	1	0	2	1	1	1	2	1	2	2	0

Note. See Table S3 for the corresponding response probabilities.

Table S11

Response frequencies of subject KS in Experiment 1 as a function of practice

Sessions	Stimuli	Response	Stimulus-onset difference d [ms]																								
			−200	−183	−167	−150	−133	−117	−100	−83	−67	−50	−33	−17	0	17	33	50	67	83	100	117	133	150	167	183	200
1–5	Intramodal	R_{tb}	0	0	0	0	0	0	0	0	0	0	2	4	8	17	39	68	88	96	98	98	98	97	98	98	97
		R_{si}	0	0	0	0	0	0	1	1	2	5	22	50	63	58	50	24	8	2	0	0	0	0	0	0	0
		R_{bt}	98	98	98	98	98	98	97	96	96	93	72	43	24	21	4	4	1	0	0	0	0	0	0	0	0
	Intermodal	R_{va}	7	0	5	4	11	8	5	17	17	19	13	21	21	21	26	22	37	42	42	42	52	55	66	67	71
		R_{si}	17	22	15	37	34	48	58	60	67	69	73	71	74	72	68	64	57	50	51	49	42	35	25	27	25
		R_{av}	69	72	73	53	51	38	33	16	13	8	10	2	1	2	1	5	2	4	3	5	1	4	3	4	0
6–10	Intramodal	R_{tb}	0	0	0	0	0	0	0	0	0	0	1	0	0	3	18	48	86	96	100	99	100	100	99	100	97
		R_{si}	0	0	0	0	1	0	0	0	8	16	65	93	99	95	79	47	9	3	0	0	0	0	0	0	0
		R_{bt}	98	99	9	100	97	100	100	99	89	81	31	7	1	1	0	0	0	0	0	0	0	0	0	0	0
	Intermodal	R_{va}	2	1	1	6	2	16	7	14	13	11	14	21	12	13	23	26	28	33	44	51	54	63	62	70	70
		R_{si}	6	13	15	24	37	43	61	66	71	77	81	76	80	76	73	67	69	64	55	41	41	30	32	26	27
		R_{av}	91	83	82	65	52	38	26	18	10	7	1	1	1	0	2	3	1	2	0	0	2	3	1	3	1
11–15	Intramodal	R_{tb}	0	0	0	1	0	0	0	0	0	0	1	0	0	6	19	71	87	97	99	99	97	100	100	100	100
		R_{si}	0	0	0	0	0	0	0	0	3	10	58	91	100	92	77	27	11	3	0	0	0	0	0	0	0
		R_{bt}	100	100	100	99	100	100	100	100	95	90	39	8	0	0	1	0	0	0	0	0	0	0	0	0	0
	Intermodal	R_{va}	0	0	0	0	3	3	8	9	17	17	13	14	14	18	18	23	22	36	42	47	47	61	58	74	63
		R_{si}	10	10	27	30	37	47	54	63	60	68	81	78	79	79	77	72	74	61	57	46	47	35	35	19	32
		R_{av}	89	86	71	63	55	44	34	24	15	3	2	0	2	0	1	1	0	0	0	0	0	0	0	1	1
16–20	Intramodal	R_{tb}	0	0	0	0	0	0	0	0	0	0	0	1	0	4	18	72	92	98	100	100	100	99	99	100	100
		R_{si}	0	0	0	0	0	0	0	0	1	6	50	81	97	95	82	26	8	1	0	0	0	0	0	0	0
		R_{bt}	100	99	99	100	100	100	100	99	98	91	49	14	2	1	0	0	0	0	0	0	0	0	0	0	0
	Intermodal	R_{va}	2	0	0	0	0	1	4	2	2	6	8	8	5	10	15	13	27	30	30	47	66	60	74	86	82
		R_{si}	9	13	21	26	34	44	56	71	79	87	86	90	92	89	84	84	70	66	68	51	30	33	24	12	15
		R_{av}	83	84	74	70	58	47	30	19	16	5	1	1	1	0	0	0	1	0	0	0	0	1	0	0	1

Note. See Table S4 for the corresponding response probabilities.

Table S12

Response frequencies of subject WF in Experiment 1 as a function of practice

Sessions	Stimuli	Response	Stimulus-onset difference d [ms]																							
			−200	−183	−167	−150	−133	−117	−100	−83	−67	−50	−33	−17	0	17	33	50	67	83	100	117	133	150	167	183	200
1–5	Intramodal	R_{tb}	0	0	0	0	0	0	0	0	1	0	9	12	15	37	64	91	97	97	97	97	98	98	98	98	98
		R_{si}	0	0	0	0	0	0	1	0	0	10	38	61	66	55	29	6	0	1	1	1	0	0	0	0	0
		R_{bt}	98	97	97	98	98	98	97	98	97	88	51	25	17	6	5	1	1	0	0	0	0	0	0	0	0
	Intermodal	R_{va}	0	0	0	4	3	1	3	6	4	4	7	6	11	16	17	23	28	37	54	62	76	84	81	88	92
		R_{si}	9	15	22	18	33	25	47	42	46	58	60	64	66	62	66	59	60	54	40	30	17	10	13	5	3
		R_{av}	88	83	76	75	60	72	48	50	47	35	30	28	21	20	15	15	10	7	4	6	5	4	3	4	3
6–10	Intramodal	R_{tb}	0	0	0	1	0	0	0	0	0	0	0	0	2	5	38	74	96	99	100	100	100	100	99	100	100
		R_{si}	0	0	0	0	0	0	0	0	1	14	50	81	94	93	59	26	2	1	0	0	0	0	0	0	0
		R_{bt}	98	99	100	98	100	100	100	100	99	86	50	19	3	2	3	0	2	0	0	0	0	0	0	0	0
	Intermodal	R_{va}	1	0	0	0	1	0	0	1	0	1	4	0	2	3	0	14	16	23	41	66	72	86	85	96	96
		R_{si}	7	9	10	24	18	18	34	45	46	47	50	74	80	81	83	82	82	76	57	34	28	13	15	4	3
		R_{av}	91	91	90	75	80	82	65	54	54	52	46	26	18	16	17	4	2	1	2	0	0	1	0	0	1
11–15	Intramodal	R_{tb}	0	0	0	0	0	0	0	0	0	0	1	1	7	21	52	87	100	99	99	100	100	100	100	100	100
		R_{si}	0	0	0	0	0	0	0	0	0	1	9	42	77	70	38	5	0	0	0	0	0	0	0	0	0
		R_{bt}	100	100	99	100	98	100	99	97	100	99	89	57	15	8	10	8	0	0	0	0	0	0	0	0	0
	Intermodal	R_{va}	0	0	0	0	0	0	1	1	3	7	8	11	16	13	28	36	46	53	57	63	76	80	89	95	93
		R_{si}	7	4	5	13	7	9	23	34	38	48	61	53	61	68	62	57	44	47	40	35	24	19	10	5	7
		R_{av}	92	95	95	87	93	91	76	65	56	44	29	36	21	19	10	7	10	0	1	1	0	1	1	0	0
16–20	Intramodal	R_{tb}	0	0	0	0	0	0	0	0	0	0	1	3	12	28	68	91	100	100	99	100	98	99	99	99	99
		R_{si}	0	0	0	0	0	0	0	0	0	0	1	8	25	34	12	0	0	0	0	0	0	0	0	0	0
		R_{bt}	99	99	99	100	98	99	98	99	100	100	98	87	63	38	20	8	0	0	0	0	0	0	0	0	0
	Intermodal	R_{va}	1	0	0	0	0	2	5	7	15	17	26	34	31	58	61	63	75	83	87	92	93	97	98	97	97
		R_{si}	0	0	0	0	0	0	2	3	3	8	14	14	35	21	19	25	17	15	8	6	1	1	0	0	0
		R_{av}	98	97	99	99	98	96	93	88	81	75	59	50	33	20	17	8	8	2	3	1	4	1	1	0	2

Note. See Table S5 for the corresponding response probabilities.

Table S13

Response frequencies of subject PK in Experiment 1 as a function of practice

Sessions	Stimuli	Response	Stimulus-onset difference d [ms]																								
			−200	−183	−167	−150	−133	−117	−100	−83	−67	−50	−33	−17	0	17	33	50	67	83	100	117	133	150	167	183	200
1–5		R_{av}	100	97	98	96	90	91	89	82	67	38	31	20	6	4	6	3	1	2	0	0	0	0	0	1	0
	Intramodal	R_{tb}	0	0	0	0	0	0	0	1	0	0	2	1	0	10	42	81	98	98	98	98	98	98	98	98	98
		R_{si}	0	0	0	0	0	0	0	0	1	10	42	84	95	87	50	16	0	0	0	0	0	0	0	0	0
		R_{bt}	98	98	98	98	98	98	98	97	97	88	54	13	3	1	5	1	0	0	0	0	0	0	0	0	0
	Intermodal	R_{va}	3	3	4	2	9	12	14	13	17	12	9	11	21	28	23	32	46	62	77	82	92	95	95	93	97
		R_{si}	1	2	1	3	8	9	14	30	36	63	74	78	71	65	69	61	48	33	17	14	6	2	3	3	0
		R_{av}	93	93	93	92	81	77	69	54	45	22	15	9	6	5	4	2	4	3	4	2	0	0	0	1	1
6–10	Intramodal	R_{tb}	0	0	1	0	0	0	0	0	0	0	0	0	2	8	46	90	100	100	100	100	100	100	100	100	100
		R_{si}	0	0	0	0	0	0	0	0	0	3	20	66	91	90	54	10	0	0	0	0	0	0	0	0	0
		R_{bt}	99	100	99	100	100	100	100	100	100	97	80	34	7	1	0	0	0	0	0	0	0	0	0	0	0
	Intermodal	R_{va}	1	0	5	0	4	6	6	8	18	14	21	20	23	25	39	38	62	78	75	93	98	97	99	99	100
		R_{si}	0	0	4	5	4	17	23	32	43	57	62	72	73	70	59	60	37	20	23	7	2	3	1	0	0
		R_{av}	99	100	91	95	92	77	71	60	39	29	17	7	3	4	2	2	1	1	2	0	0	0	0	1	0
11–15	Intramodal	R_{tb}	0	0	0	0	0	0	0	0	0	0	0	1	0	8	53	98	100	99	100	100	100	100	99	100	100
		R_{si}	0	0	0	0	0	0	0	0	0	0	11	57	94	86	47	2	0	0	0	0	0	0	0	0	0
		R_{bt}	100	100	100	100	100	100	100	100	100	100	88	42	6	4	0	0	0	0	0	0	0	0	0	0	0
	Intermodal	R_{va}	0	1	0	1	0	0	1	5	3	9	12	10	9	15	27	36	52	79	80	93	96	98	100	99	98
		R_{si}	1	2	0	1	7	12	14	33	47	54	63	82	83	76	71	58	47	20	20	7	4	1	0	0	1
		R_{av}	98	97	100	98	93	88	85	62	50	37	25	8	8	9	2	5	1	1	0	0	0	1	0	0	0
16–20	Intramodal	R_{tb}	0	0	0	0	0	1	0	0	0	0	1	0	1	9	52	93	98	99	100	100	100	99	100	100	100
		R_{si}	0	0	0	0	0	0	0	0	0	0	16	69	95	91	48	7	1	0	0	0	0	0	0	0	0
		R_{bt}	100	100	100	100	100	99	100	100	100	100	83	31	3	0	0	0	0	0	0	0	0	1	0	0	0
	Intermodal	R_{va}	0	0	1	1	0	1	2	1	1	9	3	6	14	16	17	24	47	67	76	90	92	96	97	98	100
		R_{si}	0	3	1	3	10	8	9	16	32	53	66	74	80	80	77	73	51	30	23	10	7	4	3	1	0
		R_{av}	100	97	98	96	90	91	89	82	67	38	31	20	6	4	6	3	1	2	0	0	0	0	0	1	0

Note. See Table S6 for the corresponding response probabilities.

Experiment 2

Table S14 on the next page contains the observed responses frequencies in Experiment 2 pooled across the five sessions for each subject.

Table S14

Response frequencies in Experiment 2

Subject	Intensity	Response	Stimulus-onset difference d [ms]														
			−350	−300	−250	−200	−150	−100	−50	0	50	100	150	200	250	300	350
ER	Strong	R_{va}	1	1	0	7	12	9	21	16	20	36	84	104	120	133	142
		R_{si}	8	9	23	35	59	114	110	128	126	112	61	47	28	16	16
		R_{av}	159	157	145	126	97	44	37	24	20	20	23	17	20	19	10
	Weak	R_{va}	9	9	10	14	21	22	17	18	22	34	51	65	86	104	114
		R_{si}	22	40	49	54	73	102	115	117	123	107	91	81	59	44	41
		R_{av}	135	119	108	99	74	44	36	33	23	26	26	21	23	20	11
LD	Strong	R_{va}	0	2	1	3	0	0	3	1	5	15	66	119	144	162	176
		R_{si}	4	12	33	86	122	163	170	169	167	160	111	59	33	16	2
		R_{av}	173	163	143	88	56	14	5	8	5	3	1	0	1	0	0
	Weak	R_{va}	3	0	3	1	2	5	9	7	15	24	54	83	134	153	170
		R_{si}	29	54	76	104	134	158	162	161	160	151	122	93	43	24	7
		R_{av}	145	122	98	72	40	14	6	8	3	2	2	1	1	1	1
VS	Strong	R_{va}	0	0	0	1	0	2	9	4	36	131	164	170	175	173	173
		R_{si}	0	0	0	2	5	24	115	167	133	44	11	4	0	1	3
		R_{av}	175	176	175	173	170	150	52	5	7	1	1	2	1	2	0
	Weak	R_{va}	1	0	2	2	1	8	20	28	37	117	153	173	172	175	176
		R_{si}	0	0	0	2	8	24	92	143	135	58	17	2	4	0	0
		R_{av}	174	175	174	172	166	143	63	5	3	1	6	1	0	1	0
CL	Strong	R_{va}	1	1	2	4	1	1	7	9	43	102	137	154	166	165	169
		R_{si}	11	13	31	75	133	161	160	159	123	59	34	16	7	7	7
		R_{av}	157	158	136	90	41	9	5	4	3	6	3	3	2	1	1
	Weak	R_{va}	1	1	0	5	2	3	5	15	31	78	120	145	164	160	165
		R_{si}	28	57	79	119	148	160	151	148	119	78	41	26	8	9	7
		R_{av}	136	102	85	39	19	7	5	3	5	2	5	2	1	1	0
ML	Strong	R_{va}	0	2	1	3	1	4	4	10	37	87	113	130	146	156	158
		R_{si}	4	3	4	19	39	43	72	93	110	68	43	36	18	14	9
		R_{av}	173	170	170	154	134	127	100	73	29	21	18	12	12	6	8
	Weak	R_{va}	4	3	4	3	9	9	16	29	48	74	97	106	119	137	144
		R_{si}	11	14	23	25	38	58	79	98	97	80	62	54	41	25	22
		R_{av}	157	157	150	148	124	107	80	49	28	20	19	14	16	10	8
MM	Strong	R_{va}	3	7	8	7	3	4	0	0	1	7	22	37	56	69	107
		R_{si}	33	44	68	103	140	157	173	172	167	165	144	117	98	73	48
		R_{av}	142	127	99	65	35	16	5	6	9	4	10	23	22	32	19
	Weak	R_{va}	5	5	5	5	2	3	0	2	2	4	4	13	19	30	41
		R_{si}	50	79	104	135	161	165	173	174	174	171	173	162	154	141	124
		R_{av}	122	91	66	37	13	10	4	2	1	2	1	3	4	4	13
FL	Strong	R_{va}	1	0	3	5	13	14	25	37	61	89	89	107	120	126	123
		R_{si}	20	34	58	83	111	132	137	128	110	77	82	63	49	48	49
		R_{av}	156	143	116	89	50	27	8	11	5	8	5	6	8	3	3
	Weak	R_{va}	4	8	14	17	24	17	35	46	64	55	86	76	80	73	76
		R_{si}	42	62	83	114	134	150	132	125	102	112	83	96	88	101	97
		R_{av}	131	105	80	45	18	8	6	3	6	6	6	2	6	1	2
RZ	Strong	R_{va}	0	1	2	4	3	11	15	17	21	64	132	157	175	174	170
		R_{si}	4	4	15	30	60	122	157	160	155	114	45	20	2	3	7
		R_{av}	172	168	161	144	115	44	6	1	2	0	1	1	1	1	1
	Weak	R_{va}	0	0	0	4	8	12	15	23	30	55	109	141	160	165	169
		R_{si}	4	2	10	21	39	93	150	150	147	121	67	34	16	10	7
		R_{av}	174	175	167	153	130	72	13	4	1	1	2	2	2	3	2
LF	Strong	R_{va}	10	16	11	28	38	42	60	58	51	62	66	75	80	72	98
		R_{si}	30	37	49	74	94	114	110	115	122	112	106	98	92	104	72
		R_{av}	134	120	113	69	43	15	4	2	1	0	2	2	2	0	2
	Weak	R_{va}	2	5	1	4	6	9	10	16	17	16	16	15	22	18	19
		R_{si}	79	89	95	120	130	153	157	151	154	155	155	154	150	154	156
		R_{av}	93	78	80	44	31	11	9	4	2	2	2	3	2	2	1
SE	Strong	R_{va}	2	0	2	2	1	0	1	2	8	25	54	119	149	170	172
		R_{si}	3	10	24	62	121	160	174	172	168	146	113	55	26	5	3
		R_{av}	170	166	150	112	53	16	1	2	0	4	8	2	1	1	1
	Weak	R_{va}	0	1	1	2	2	3	6	5	2	29	76	134	158	167	170
		R_{si}	1	6	16	31	90	140	163	167	170	146	97	39	15	6	4
		R_{av}	174	167	159	143	84	32	7	3	4	1	2	2	3	3	1

Note. See Table S7 for the corresponding response probabilities.

Tabular representation of the proportions of corrected/excluded responses

Tables S15 and S16 contain the individual percentages of finger errors (response corrections), lapses (double-clicks), premature responses (RTs < 150 ms), and delayed responses (RTs > 5,000 ms) in Experiments 1 and 2.

Experiment 1

Table S15

Individual percentages of response corrections, lapses, premature responses, and delayed responses in Experiment 1

Subject	Correction	Lapse	Premature	Delayed
AN	< 0.1	0.1	0.1	< 0.1
MS	3.2	0.3	0.8	0.2
KS	0.1	0.5	< 0.1	0.2
WF	< 0.1	0.1	< 0.1	0.2
PK	1.9	0.1	< 0.1	0.1

Experiment 2

Table S16

Individual percentages of response corrections, lapses, premature responses, and delayed responses in Experiment 2

Subject	Correction	Lapse	Premature	Delayed
ER	1.7	0.1	0.1	0
LD	0.1	0.3	< 0.1	< 0.1
VS	0.4	< 0.1	0.1	0.1
CL	1.6	4.8	0.2	0.2
ML	0.3	0.8	0	0.6
MM	2.6	0.6	0.1	0.1
FL	< 0.1	1.5	< 0.1	0.1
RZ	0.3	0.2	0.1	< 0.1
LF	0.2	2.3	0	0.4
SE	1.1	0.2	< 0.1	0

Graphical representation of the two-threshold model fits

Experiment 1

Figures S1–6 show the fits of the two-threshold model to the individual psychometric functions from Experiment 1 pooled across all 20 sessions (Figure S1) and divided into four practice levels (Figures S2, S3, S4, S5, and S6 for subjects AN, MS, KS, WF, and PK, respectively).

For intramodal stimuli, the judgment *tb* (*bt*) indicates that the top stimulus appeared before (after) the bottom stimulus. For intermodal stimuli, the judgment *va* (*av*) indicates that the visual stimulus appeared before (after) the auditory stimulus. Error bars reflect bootstrapped 95% confidence intervals.

Figure S1

Fits of the two-threshold model to the psychometric functions observed in Experiment 1 pooled across all 20 sessions

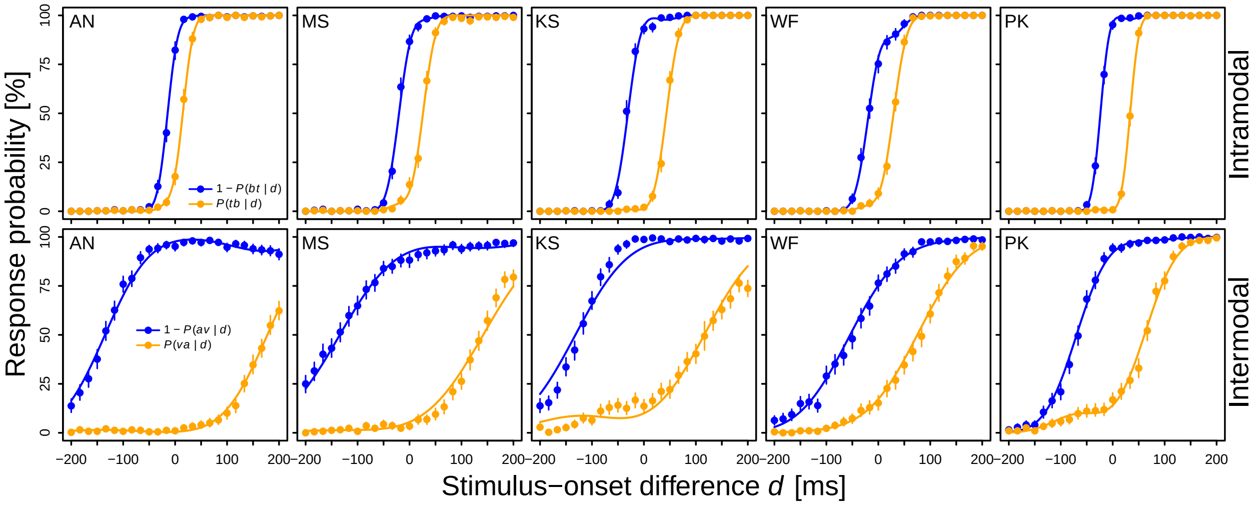

Figure S2

Fits of the two-threshold model to the psychometric functions from subject AN in Experiment 1 divided into four practice levels

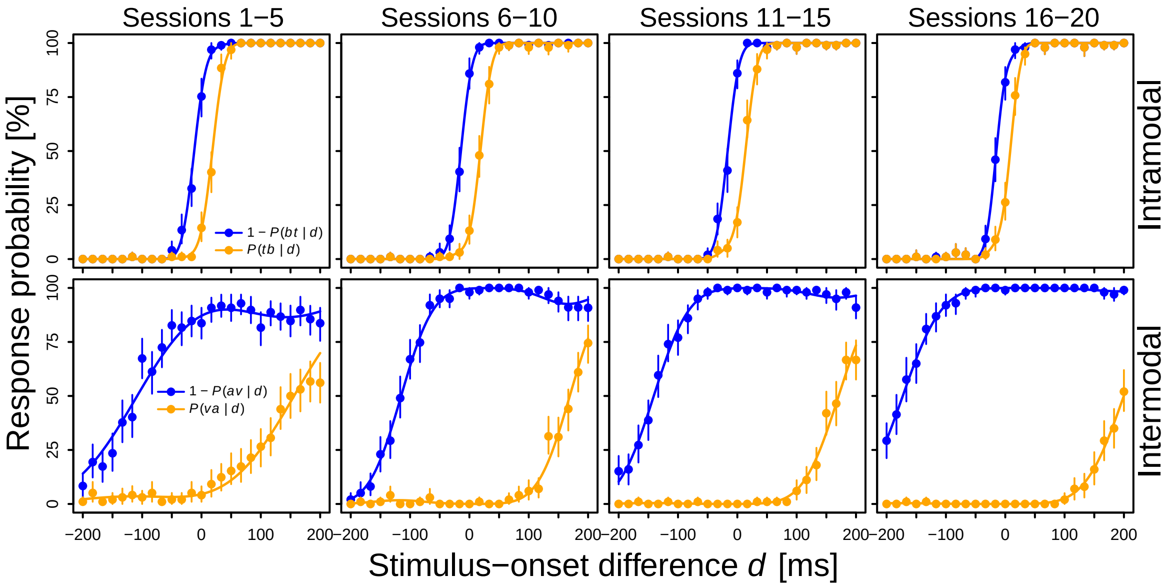

Figure S3

Fits of the two-threshold model to the psychometric functions from subject MS in Experiment 1 divided into four practice levels

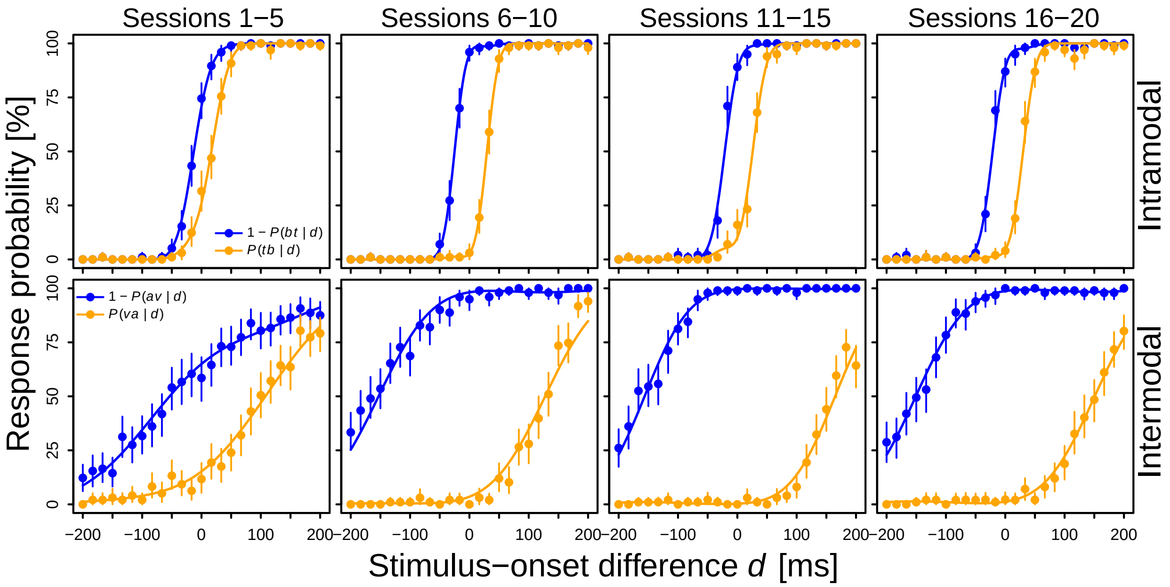

Figure S4

Fits of the two-threshold model to the psychometric functions from subject MS in Experiment 1 divided into four practice levels

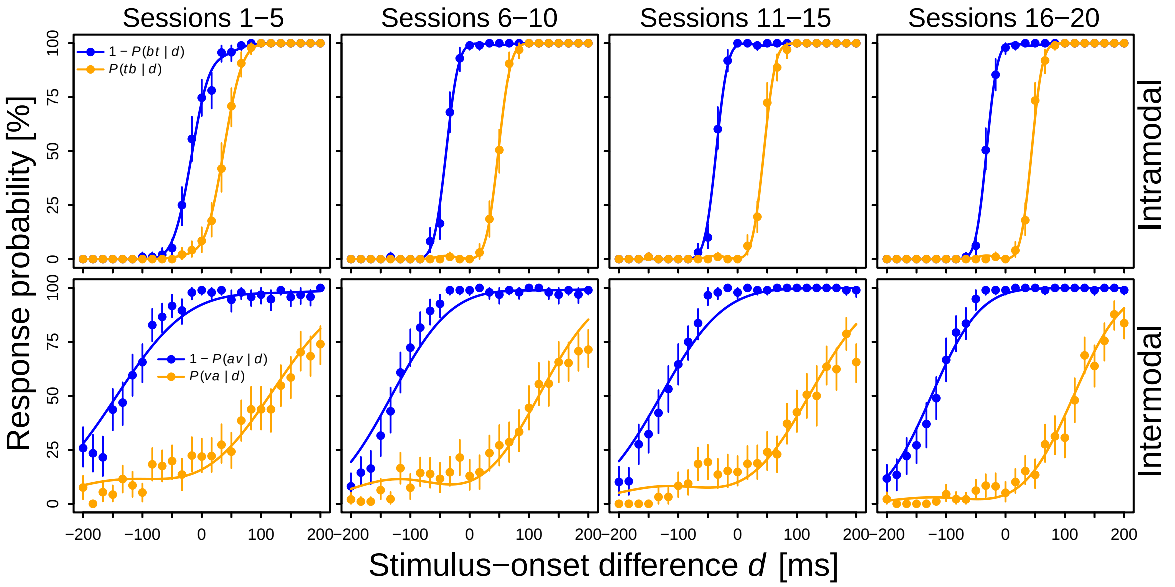

Figure S5

Fits of the two-threshold model to the psychometric functions from subject WF in Experiment 1 divided into four practice levels

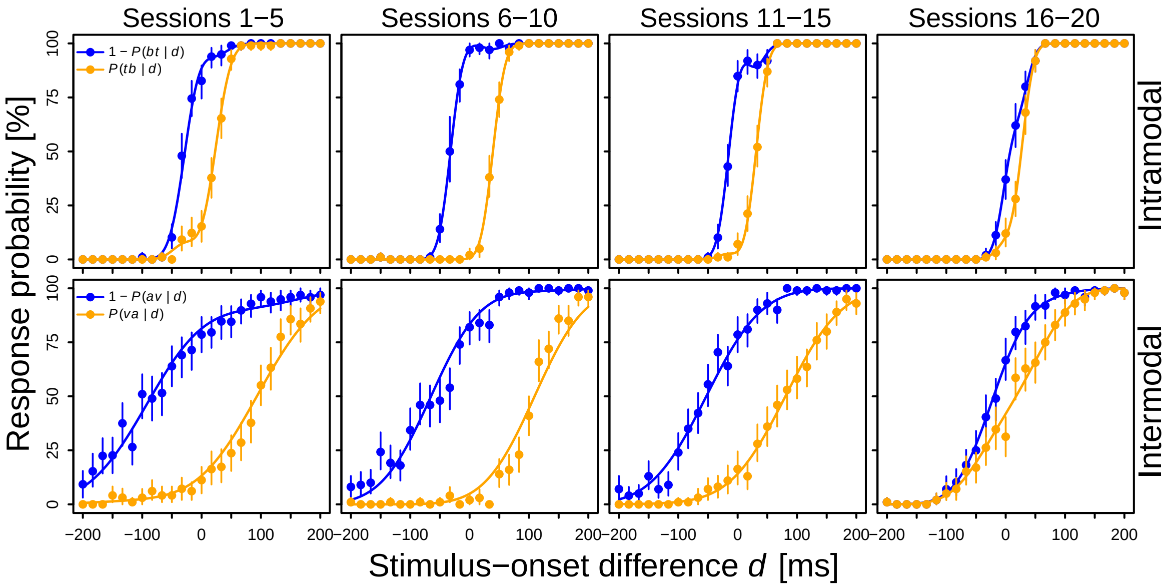

Figure S6

Fits of the two-threshold model to the psychometric functions from subject PK in Experiment 1 divided into four practice levels

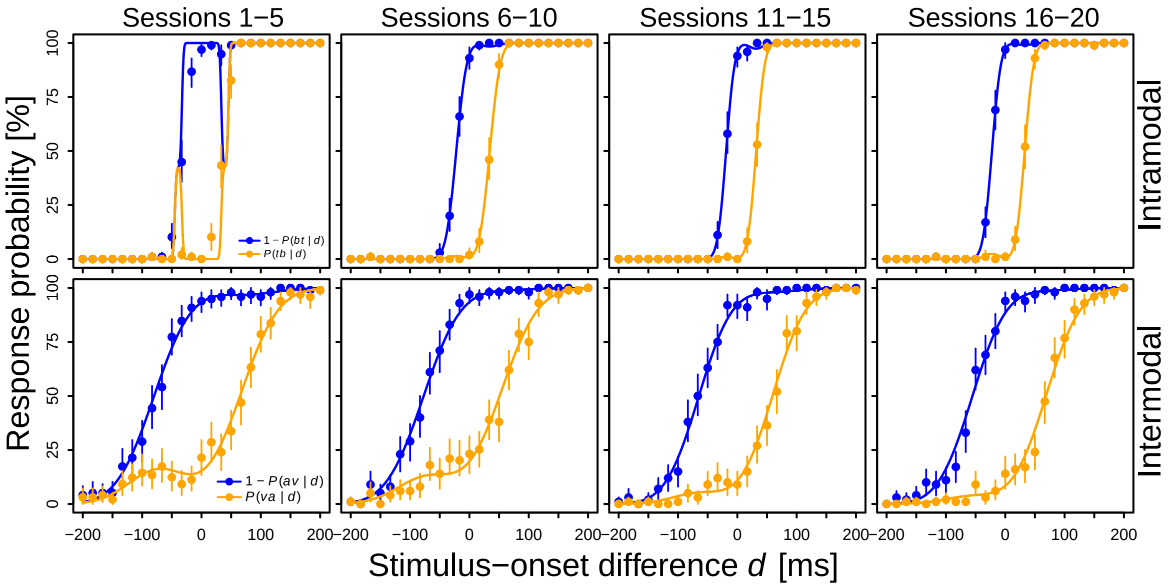

Experiment 2

Figure S7 shows the fits of two-threshold model to the individual psychometric functions from Experiment 2.

Figure S7

Fits of the two-threshold model to the psychometric functions observed in Experiment 2 pooled across all five sessions

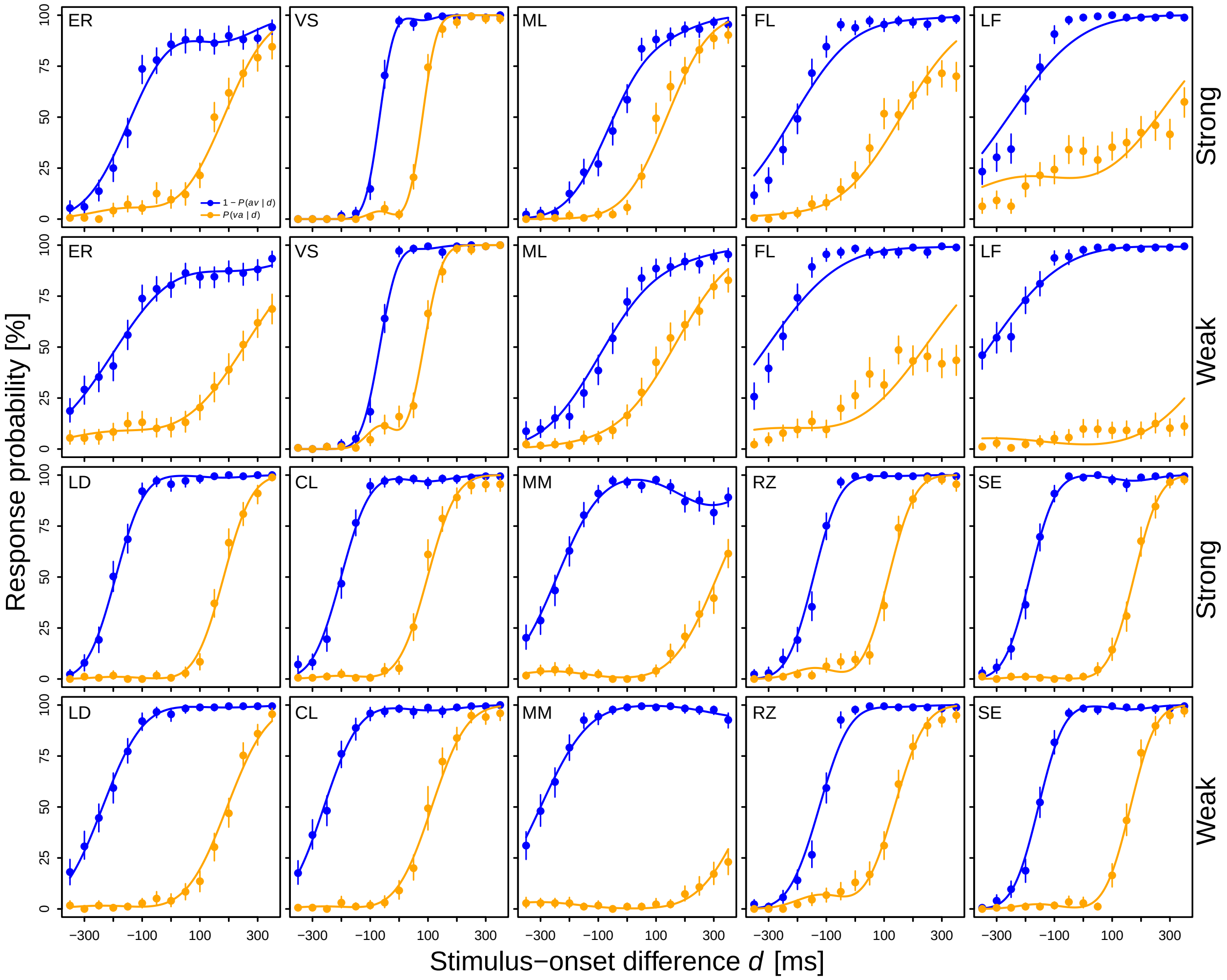

Supplement: Supplementary file 1 — (pdf 701 KB) [file 13423_2025_2797_MOESM1_ESM.pdf]
